# Modeling the Architecture of Depolymerase-Containing Receptor Binding Proteins in *Klebsiella* Phages

**DOI:** 10.3389/fmicb.2019.02649

**Published:** 2019-11-15

**Authors:** Agnieszka Latka, Petr G. Leiman, Zuzanna Drulis-Kawa, Yves Briers

**Affiliations:** ^1^Laboratory of Applied Biotechnology, Department of Biotechnology, Ghent University, Ghent, Belgium; ^2^Department of Pathogen Biology and Immunology, Institute of Genetics and Microbiology, University of Wrocław, Wrocław, Poland; ^3^Sealy Center for Structural Biology and Molecular Biophysics, Department of Biochemistry and Molecular Biology, The University of Texas Medical Branch, Galveston, TX, United States

**Keywords:** horizontal transfer, tail fiber genes, receptor binding protein, phage evolution, depolymerase

## Abstract

*Klebsiella pneumoniae* carries a thick polysaccharide capsule. This highly variable chemical structure plays an important role in its virulence. Many *Klebsiella* bacteriophages recognize this capsule with a receptor binding protein (RBP) that contains a depolymerase domain. This domain degrades the capsule to initiate phage infection. RBPs are highly specific and thus largely determine the host spectrum of the phage. A majority of known *Klebsiella* phages have only one or two RBPs, but phages with up to 11 RBPs with depolymerase activity and a broad host spectrum have been identified. A detailed bioinformatic analysis shows that similar RBP domains repeatedly occur in *K. pneumoniae* phages with structural RBP domains for attachment of an RBP to the phage tail (anchor domain) or for branching of RBPs (T4gp10-like domain). Structural domains determining the RBP architecture are located at the N-terminus, while the depolymerase is located in the center of protein. Occasionally, the RBP is complemented with an autocleavable chaperone domain at the distal end serving for folding and multimerization. The enzymatic domain is subjected to an intense horizontal transfer to rapidly shift the phage host spectrum without affecting the RBP architecture. These analyses allowed to model a set of conserved RBP architectures, indicating evolutionary linkages.

## Introduction

*Klebsiella pneumoniae* is a Gram-negative bacillus. In spite of being part of the natural human and animal flora, *K. pneumoniae* is also the widespread cause of both nosocomial and community acquired infections. Since 2013 *K. pneumoniae* has been marked as a prominent member of the carbapenem-resistant *Enterobacteriaceae* (CRE), featured by a multidrug-resistant phenotype and labeled as a class of antibiotic-resistant bacteria for which novel ways of therapy are most urgent (Weiner et al., 2016; Calfee, 2017). As natural bacterial predators, bacteriophages have since long been proposed as promising alternatives to antibiotic therapy. The large majority of phages is highly specific with a host spectrum defined at the species/strain level. This high specificity necessitates the selection of a phage sur-mesure for a personalized treatment or the use of a phage cocktail that covers a broader host range. Major determinants of host specificity are the phage receptor binding proteins (RBPs) that mediate the initial contact with the receptor on the host cell envelope (Williams et al., 2008). This initial contact can be based on a direct binding of long tail fibers or shorter tailspikes to the cell surface receptor. Some RBPs possess a depolymerase activity to degrade bacterial exopolysaccharides comprising the capsule (CPS), lipopolysaccharides (LPS) or biofilm matrix (Majkowska-Skrobek et al., 2016, 2018; Olszak et al., 2017). Interaction of RBPs with their cell wall receptors are essential to initiate the infection process (Andres et al., 2010; Broeker et al., 2018).

The primary receptor targeted by RBPs of many *Klebsiella* specific phages is the thick polysaccharide capsule, which is a hallmark feature of *K. pneumoniae.* The capsule is a crucial virulence factor as it forms a physical barrier to some antibiotics and host immune mechanisms, enabling bacteria to avoid phagocytosis or complement-mediated killing (Cortés et al., 2002; Lee et al., 2017; Majkowska-Skrobek et al., 2018). Differences in sugar composition, the specific ratio of various sugar components as well as variation in the locus organization are the base to distinguish at least 79 capsular serotypes called K antigens and 134 capsular loci (KL) among *Klebsiella* species (Pan et al., 2015; Wyres et al., 2016; Wick et al., 2018). This capsular diversity correlates to a correspondingly high variation of *Klebsiella* phage RBPs that contain a specific polysaccharide-depolymerizing domain (Schmid et al., 2015; Latka et al., 2017). Such domains cleave the O-glycosidic bond of capsular polysaccharides following either a hydrolase or a lyase mechanism. Hydrolases (e.g., sialidases, rhamnosidases, levanases, dextranases, and xylanases) involve a water molecule for cleavage, whereas lyases [e.g., hyaluronate lyases (hyaluronidases), pectin/pectate lyases, alginate lyases, K5 lyases] cleave by β-elimination with introduction of new double bond (Davies and Henrissat, 1995; Sutherland, 1995; Pires et al., 2016). In spite of a high diversity in enzyme specificity and primary amino acid sequence, many known depolymerases contain an elongated, highly interwoven β-helical domain that forms the specific catalytic pocket. In addition, this β-helical domain contributes to a high protein stability in harsh environments (Yan et al., 2014; Majkowska-Skrobek et al., 2016). An overview of (experimentally confirmed) RBPs with depolymerase activity has been recently reported (Latka et al., 2017).

**FIGURE 1:**
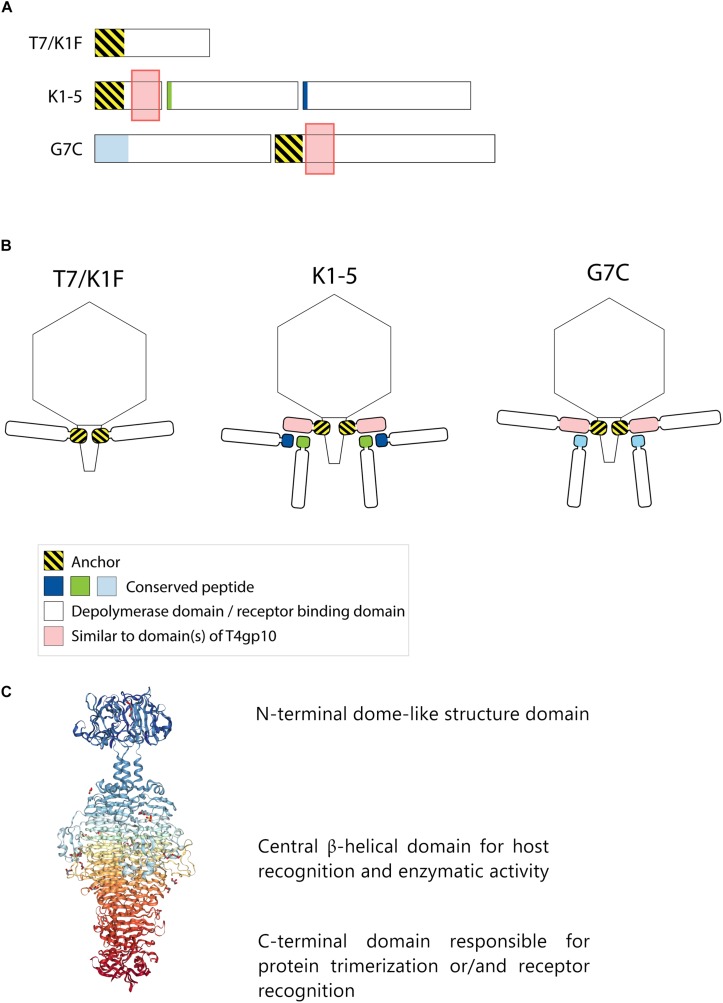
Anchor and anchor-branched receptor binding protein (RBP) complexes confirmed by structural experiments. **(A)** The modular genetic organization of RBPs in single (T7 and K1F) and double RBP systems (K1-5 and G7C phages). **(B)** Schematic modeling of four different RBP systems in the virion structure. The T7 tail fiber (gene 17, T7p52) and K1F tail fiber (gene 17, CKV1F_gp36) have only an N-terminal anchor domain; K1-5 uses an adapter protein (gp37 with T4gp10-like domain) interacting with K5 lyase (gp46) and K1 endosialidase (gp47) *via* a conserved hepta- and undecapeptide, respectively; Phage G7C produces an anchor-branched complex with one anchored RBP (gp66) having a T4gp10-like domain and the second RBP connected *via* a conserved peptide to theT4gp10-like domain. **(C)** Modular structure of the model tail spike of *Salmonella* phage P22 (PDB ID 2XC1), illustrating a typical modular structure of RBPs. A N-terminal dome-like structure domain, a central β-helical domain for host recognition and enzymatic activity and a C-terminal domain responsible for protein trimerization or/and receptor recognition are shown (Berman et al., 2000; Seul et al., 2014; Rose et al., 2018).

Receptor binding protein with depolymerase activity have a modular structure with the enzymatic domain located in the central part (Figure 1C). The C-terminus of the RBP may comprise a chaperone that assists in a proper folding and trimerization followed by autoproteolytic removal or an additional domain involved in host cell recognition (Weigele et al., 2003; Cornelissen et al., 2011; Schwarzer et al., 2012; Seul et al., 2014; Yan et al., 2014). Autocleavage of the C-terminal chaperone was also reported as a common feature among endosialidases and other tail spikes and tail fibers, necessary to increase the unfolding barrier and to trap the mature trimer in a more kinetically stable conformation (Schwarzer et al., 2007). The N-terminal dome-like domain attaches the RBP to the phage particle by a flexible connector. A modular architecture of RBPs allows for rapid evolution via horizontal gene transfer leading to host range modification. Whereas structural domains responsible for attachment to the tail apparatus are repeatedly present in many phylogenetically related phages, the domains for host cell receptor recognition/degradation are subjected to intense exchanges across phylogenetic borders. In addition, the latter RBP domains undergo further constant modification through vertical transfer and accumulation of mutations (Stummeyer et al., 2006; Barbirz et al., 2008; Leiman and Molineux, 2008; Schwarzer et al., 2012; Latka et al., 2017). The tail fibers of *E. coli* phage T7 and its relative K1F are type examples of a horizontal transfer of the C-terminal RBP domain. These tail fibers share a conserved N-terminal domain of ∼140 resides that anchors the tail fiber to the phage particle (Figure 1). However, T7 has a C-terminal domain that recognizes and binds lipopolysaccharide, whereas K1F produces an endosialidase specific for recognition and cleavage of *E. coli* K1 capsular polysaccharide (Steven et al., 1988; Stummeyer et al., 2005).

Phages with a single RBP such as T7 and K1F are most frequently described in the literature. However, several phages belonging to *Podoviridae* have also acquired two different RBPs corresponding to a dual receptor-specificity. E.g., *K. pneumoniae* podoviruses K5-2, K5-4, and KP32 possess two RBPs with a depolymerase domain with different enzymatic specificity (Hsieh et al., 2017; Majkowska-Skrobek et al., 2018). In the last decade, an increasing body of knowledge about the genetic and structural organization of RBPs of such bispecific phages has been acquired, particularly for different T7-like phages such as K1-5 and SP6 (Stummeyer et al., 2006; Leiman et al., 2007; Gebhart et al., 2017; Tu et al., 2017). These phages use a small trimeric adapter protein of approximately 300 amino acids, sharing a high N-terminal sequence identity to T7 and K1F tail fibers (Figure 1). In addition, phage K1-5 encodes a K5 lyase (gp46) and an endosialidase (gp47), which are specific for *E. coli* K5 and K1 capsule, respectively. CryoEM studies and bioinformatics suggest that K5 lyase binds through a heptapeptide (MAKLTKP) to a specific site in the middle of the K1-5 adapter protein, whereas the second tailspike (endosialidase) binds to a different specific site in its C-terminal part through an undecapeptide (MIQRLGSSLVK) (Leiman et al., 2007). The heptapeptide, undecapeptide, and adapter sequences are conserved among other T7-like phages that infect different bacterial species and that carry two different RBPs on the phage particle (e.g., SP6), demonstrating a conserved mechanism for attachment of two RBPs (Figure 1). Notably, domains recognizing the same host receptor can have highly similar amino acid sequence but can be incorporated into a different RBP architecture. For example, the K1F and K1-5 endosialidase domains specific to K1 capsule show 72% identity with a coverage of 86%, but in phage K1F the endosialidase domain is present in a single RBP with anchor domain, whereas in phage K1-5 the endosialidase is connected to the phage particle via an intermediate adapter protein. A homolog of the endosialidase domain of podovirus K1F is also present in the multivalent *E. coli* myovirus phi92 (EndoN92; 53% identity with a coverage of 83%), demonstrating exchange of the domain across members of the *Podoviridae* and *Myoviridae* families with highly different tail structures (Schwarzer et al., 2012, 2015).

More recently, a different organization of two types of RBPs in a single phage particle has been reported based on structural, genetic and biochemical studies of the RBPs of *E. coli* N4-like podovirus G7C (Prokhorov et al., 2017). G7C carries two RBPs – a longer G7Cgp66 and a shorter G7Cgp63.1 protein. The specificity of the longer G7cgp66 protein is unknown, but the shorter G7Cgp63.1 RBP was shown to deacetylate the O-antigen of *E. coli* 4S while leaving the backbone of the sugar intact. G7Cgp63.1 does not interact with the phage particle directly. Instead, it binds to G7Cgp66, which is attached to the phage particle with its N-terminal anchor domain (Figure 1B). The gp63.1 binding region of G7Cgp66 (residues 138–294) is homologous to subdomains D2 and D3 of phage T4 gp10. In phage T4, these subdomains of gp10 serve as an attachment site for two proteins – gp11, which interacts with the long tail fiber RBP or short tail fiber RBP, depending on the state of the phage particle, and gp12, the short tail fiber RBP (Taylor et al., 2016). This protein complex represents a *bona fide* branched structure involved in the transmission of the signal of reversible host binding, culminating in irreversible binding, sheath contraction and DNA ejection. The T4gp10-like domains are prevalent in RBPs of unrelated phages across *Podoviridae* and *Myoviridae*, which may reflect its ancient evolutionary role in the transduction from reversible to irreversible binding during phage adsorption (Prokhorov et al., 2017).

Interestingly, the T4gp10-like region of G7Cgp66 covers both subdomain D2 and D3 of T4gp10 to which T4gp11 and T4gp12 are attached. Though, G7Cgp66 and G7Cgp63.1 form a 1:1 complex, suggesting that G7Cgp63.1 occupies only one of the two RBP binding sites on G7Cgp66. Notably, orthologs of G7Cgp66 in some G7C-like viruses do not contain a putative enzymatic domain but nevertheless retain the N-terminal particle-binding domain and the T4gp10-like domains. As such their attachment apparatus becomes similar to the adapter system of phage K1-5. The N-terminal part of G7Cgp63.1 that interacts with the T4gp10-like domain of G7Cgp66 is also found at the N-terminus of other tail spikes that have a branched structure, such as CBA120 phage tail spike 1 (Chen et al., 2014) and other putative tail spikes of Vil-like phages (Adriaenssens et al., 2012). CBA120 encodes four tail spikes (TSP1-4) from which two (TSP2 and TSP4) are equipped with T4gp10-like domains D2 and D3. These domains provide side or off-axis attachment sites for TSP1 and TSP3. The conserved N-terminal part of TSP4 attaches the whole branched structure composed of four TSPs to the baseplate of the virion (Plattner et al., 2019).

*Klebsiella* jumbo viruses may also have a multitude of RBPs resulting accordingly in a broader host spectrum. The highest variation of depolymerases has been described for the jumbo ΦK64-1 phage, which is able to infect *K. pneumoniae* of 10 different capsular serotypes and for which 11 different polysaccharide depolymerases have been identified (Pan et al., 2017). Also, the jumbo vB_KleM-RaK2 phage encodes a multitude of putative depolymerases (Simoliūnas et al., 2013). Electron microscopy images of such jumbo phages typically show an elaborated tail fiber apparatus with a high structural complexity, but for which structural insights are currently lacking.

In this study we present an extensive bioinformatic analysis of the structural and genetic organization of depolymerase-containing RBPs in *Klebsiella* phages. Next-generation sequencing technologies have recently led to a large number of sequenced phage genomes in public databases including *Klebsiella* viruses (*n* = 97). In a large proportion of these phages (59/97; 61%) we could predict an RBP with depolymerase activity. The observed large diversity of depolymerase domains accommodates the high diversity of capsular serotypes among *Klebsiella* strains. Based on an integrated analysis, we propose diverse RBP architectures in *Klebsiella* phages.

## Materials and Methods

At first, *Klebsiella* phages were collected from the GenBank database (retrieved at 15.08.2018). A number of 59 phages were finally analyzed (Supplementary Table S1). From these phages proteins annotated as tail fibers or tail spikes were analyzed with BlastP^1^ (Altschul et al., 1990), Phyre2^2^ (Kelley et al., 2015), SWISS-MODEL^3^ (Bordoli et al., 2009; Bordoli and Schwede, 2012), HMMER^4^ (Finn et al., 2011) and HHPred^5^ (Zimmermann et al., 2018) to identify phages that encode RBPs with putative depolymerase activity (Supplementary Table S2). If neither a tail fiber nor a tail spike gene was found in the genome, we analyzed all genes located in the vicinity of annotated structural genes. BlastP (protein–protein Blast) was performed against the non-redundant protein sequences (nr) database using standard parameters (expect threshold: 10, word size: 6, MATRIX: BLOSUM62, Gap cost: existence 11, extension 1, conditional compositional score matrix adjustment). HMMER was used in the quick search mode against: Reference Proteomes, UniProtKB, SwissProt, and Pfam with significance E-values: 0.01 (sequence) and 0.03 (hit). For Phyre2 the normal modeling mode was used. HHPred homology detection structure prediction was run using the PDB_mmCIF70 database and the following parameters [MSA generation method: HHblits uniclust30_2018_08; Maximal no. of MSA generation steps: 3; E-value incl. threshold for MSA generation: 1e-3; minimal sequence identity of MSA hits with query (%): 0; minimal coverage of MSA hits (%) 20; Secondary structure scoring: during alignment; Alignment Mode: Realign with MAC: local:norealign; MAC realignment threshold: 0.3; No. of target sequences: 250; Min. probability in hit list (>10%): 20].

Criteria for the prediction of putative depolymerase activity were (Supplementary Table S2): (1) the protein must be longer than 200 residues; (2) the protein must be annotated as tail fiber/tail spike/hypothetical protein in the NCBI database; (3) the protein must show homology to domains annotated as lyase [hyaluronate lyases (hyaluronidases), pectin/pectate lyases, alginate lyases, K5 lyases] or hydrolase (sialidases, rhamnosidases, levanases, dextranases, and xylanases) with a confidence of at least 40% in Phyre2 or the enzymatic domain should also be recognized by at least SWISS-MODEL, HMMER, or BlastP; (4) the length of homology with one of these enzymatic domains should span at least 100 residues; (5) a typical β-helical structure should be predicted by Phyre2. These RBP depolymerases are indicated without additional labeling in the tables. Proteins possessing experimentally confirmed depolymerizing activity were marked in the tables with (a). When the RBP was only partially fulfilling the above-mentioned criteria, it was indicated with label (b). These putative depolymerases that could only be predicted with a lower probability were fulfilling criteria 1 and 2, but the confidence of the Phyre2 prediction was below 40% or only SWISS-MODEL, HMMER or BLASTP gave a positive prediction. In addition, the homologous domain only spans between 50 and100 amino acids and no β-helical structure could be predicted with Phyre2 (for details see Supplementary Table S2).

All selected *Klebsiella* phages were then grouped based on gene homology and a conserved gene synteny into KP32viruses, KP34viruses, and KP36viruses and into groups containing only *Klebsiella*-specific phages similar to phage JD001 (belonging to Jedunavirus), similar to phage Menlow (belonging to Ackermannviridae), similar to phage ΦK64-1 (belonging to Alcyoneusvirus). Within each group, further subdivisions were proposed for the purpose of this study, based on the organization of the RBP gene cluster (number of RBPs, length of different genes, presence of anchor, or branching domains).

When there was one RBP, a domain in the N-terminus of a RBP was annotated as ‘anchor’ when there was at least an identity of 39% (BLASTP) over at least 166 residues starting from the N-terminus of the corresponding protein among phages belonging to the same group. These parameters were set empirically based on the shortest identity region found among all RBPs, specifically in the first RBP of phage IL33, belonging to KP32viruses group B (166 amino acids) and the identity% of the first RBP of phage Kp1. When more than one RBP was present, the anchor domain was annotated in the RBP in which also a T4gp10-like domain was detected. In the other RBP(s) the N-terminal conserved sequence was called ‘conserved peptide,’ which was also generally shorter than the anchor domains. To define consensus sequences of the anchor domains and conserved peptides, multiple sequence or pairwise alignment were used, since these structures are highly conserved among phages from the same group. To identify domains involved in the branching of RBPs, the sequences were analyzed by HHPred performing protein structure prediction^5^ (Zimmermann et al., 2018) in search for domains homologous to T4gp10 domain 2 and 3 as experimentally confirmed attachment sites (Prokhorov et al., 2017). WebLogos of the anchor domains and conserved peptides were created with the online available tool^6^ (Crooks et al., 2004).

## Results

Taxonomically closely related phages are characterized by a synteny of conserved structural genes interrupted by divergent RBP genes, which are subject to intensive horizontal transfer. We therefore inspected the region of structural genes across different *Klebsiella* phages within specific phage genera to identify potential RBPs based on a broken synteny. Subsequently, we analyzed the presence of putative enzymatic domains within the identified RBPs. Based on homology, protein size and structure, we looked for conserved domains (anchor domain, T4gp10-like domain) that may explain the RBP architecture of the particular phage. To further refine this architecture, we analyzed the sequence for the presence of conserved peptides that may mediate attachment to putative T4gp10-like domains. We integrated all these data to model the RBP apparatus of an extensive and diverse set of *Klebsiella* phages with (predicted) depolymerase activity.

### RBPs From Selected *Klebsiella* Podoviruses

#### KP32viruses

**FIGURE 2:**
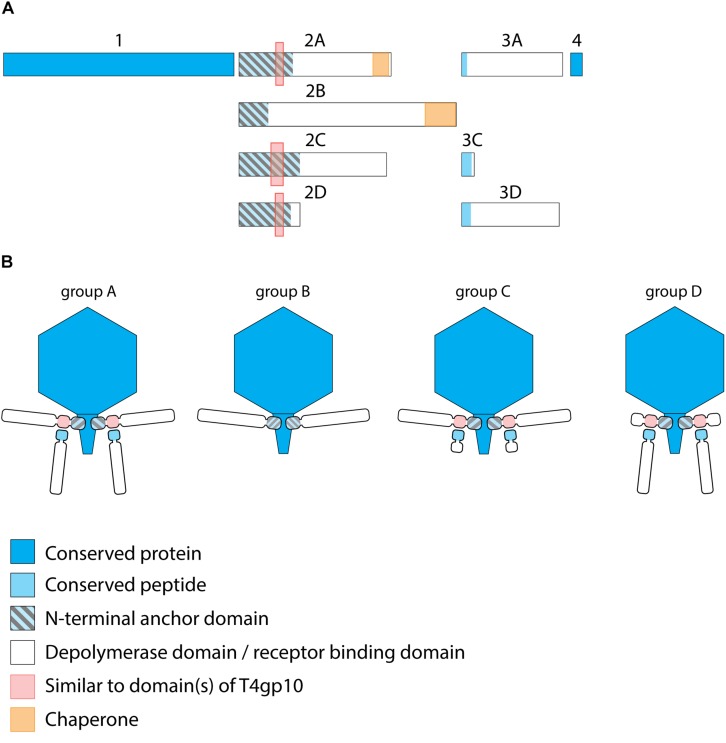
Receptor binding protein systems of KP32viruses. Phages and their RBPs that are proposed to follow these systems, including their grouping into groups A, B, C and D, are summarized in Table 1. **(A)** The modular composition of RBP genes of phages belonging to four different groups (A, B, C, and D) is shown relative to the broken gene synteny of phage KP32. For simplicity, only one flanking gene conserved across all groups is shown at each side. Annotations are given according to GenBank or according to their modeled function in this study (between brackets): Protein (1) – internal virion protein D; (2) – tail fiber protein (2A,B,C – anchor with depolymerase; 2D – anchor with truncated protein); (3) – hypothetical protein (3A,D – depolymerase with conserved peptide, 3C – truncated protein with conserved peptide); (4) – lysis protein. **(B)** Schematic models of RBP systems in phage particles of KP32viruses. Group A – two RBPs: anchor-branch attachment mode; Group B – one RBP: anchor attachment mode; Group C – two RBPs: anchor-branch attachment mode, second RBP truncated; Group D – two RBPs: anchor-branch attachment mode, first RBP truncated.

KP32viruses belong to *Podoviridae* and have tail fibers attached to a short, non-contractile tail. A similar synteny of highly conserved structural genes is observed across twenty-one KP32viruses (Supplementary Table S1A). Yet, one or two non-conserved genes of different lengths interrupt this synteny after the gene encoding the internal virion protein D. They were identified as putative RBPs and in a few cases also experimentally verified (Hsieh et al., 2017; Majkowska-Skrobek et al., 2018; Solovieva et al., 2018) (Table 1). We found four different RBP organizations (groups A, B, C, and D; Figure 2). The N-termini of the first RBPs are shared with high sequence identity (46–72%) across all KP32viruses. Specifically, residues 1–154 of the first RBPs are highly similar to the N-terminal domain of the phage T7 tail fiber (pfam03906). In group A of KP32viruses, this conserved N-terminal domain (Supplementary Figure S1A) also contains a region that is similar to a fragment of a T4gp10 branching domain, offering a potential attachment point for a secondary tail fiber. The other domain(s) of these 744–903 aa long first RBPs do not share identity with the corresponding domain of the group A model phage KP32. All central domains are predicted to possess enzymatic activity (hydrolase, lyase) but with different specificity. In addition, they all are predicted to possess a characteristic β-helical structure (Supplementary Table S2). In phage KP32, there is an additional C-terminal domain with predicted chaperone activity, which is absent in all other RBPs of the group A KP32viruses.

**TABLE 1:** Overview of RBPs of KP32viruses with (predicted) depolymerase activity grouped according to the different observed RBP systems.

**Phage**	**First RBP (protein 2,** Figure 2)						**Second RBP (protein 3,** Figure 2)					
	**Accession number**	**Number of aa**	**Alignment with first RBP from KP32**				**Accession number**	**Number of aa**	**Alignment with second RBP from KP32**			
			**Cover**	**E-value**	**Identity**	**Identity range**			**Cover**	**E-value**	**Identity**	**Identity range**
**Group A (two RBPs: anchor-branch attachment mode)**												
KP32	YP_003347555.1^a^	869	100%	0.0	100%	869/869	YP_003347556.1^a^	576	100%	0.0	100%	576/576
K11	YP_002003830.1	875	40%	5E-144	68%	241/355	YP_002003831.1	596	88%	5E-41	27%	149/543
KP32 194	AWN07125.1	777	39%	2E-139	67%	233/347	AWN07126.1	555	8%	0.0001	51%	30/59
KpV763	AOT28172.1	777	39%	2E-139	67%	232/347	AOT28173.1	524	8%	0.0001	51%	30/59
KP32 192	AWN07083.1	777	39%	5E-139	67%	232/347	AWN07084.1	555	8%	0.00006	53%	31/59
K5-2	APZ82804.1^a^	792	35%	6E-139	71%	219/307	APZ82805.1^a^	685	5%	0.00009	87%	26/30
KP32 196	AWN07213.1	903	40%	6E-134	64%	227/354	AWN07214.1	613	23%	0.00001	34%	48/143
KpV766	AOZ65569.1	903	40%	6E-133	64%	226/354	AOZ65570.1^b^	511	30%	7E-12	38%	42/111
KpV289	YP_009215498.1	903	40%	7E-133	63%	224/354	YP_009215499.1^b^	511	18%	0.0008	38%	42/111
IME 205	ALT58497.1	793	37%	7E-132	64%	209/329	ALT58498.1	641	5%	0.001	83%	24/29
K5	YP_009198668.1	817	43%	2E-131	59%	225/381	YP_009198669.1	575	99%	0.0	87%	498/575
K5-4	APZ82847.1^a^	744	44%	2E-120	55%	218/396	APZ82848.1^a^	684	5%	0.000008	88%	28/32
**Group B (one RBP: anchor attachment mode)**												
KP32 195	AWN07172.1	1017	33%	1E-51	46%	136/293	No second RBP present					
SH-Kp 152410	AUV61507.1	1017	33%	4E-50	46%	134/293						
IL33	ARB12452.1^b^	1242	33%	3E-59	72%	119/166						
PRA33	ARB12406.1^b^	1242	33%	4E-58	71%	119/168						
BIS33	ARB12500.1^b^	1242	33%	1E-57	70%	117/166						
IME321	AXE28435.1^c^	820	22%	1E-53	59%	114/194						
Kp1	YP_009190948.1^d^	1017	62%	2E-59	39%	166/422						
**Group C (two RBPs: anchor-branch attachment mode, second RBP truncated)**												
KpV767	AOZ65519.1	843 aa	39%	2E-127	61%	212/347	AOZ65520.1^c^	69 aa	9%	0.000003	51%	28/55
**Group D (two RBPs: anchor-branch attachment mode, first RBP truncated)**												
2044-307w	ASZ78307.1^c^	347 aa	33%	4E-122	65%	192/295	ASZ78308.1^b^	556 aa	8%	0.002	55%	28/51

*The different RBP systems of KP32viruses are visualized in Figure 2. ^*a*^RBP for which the depolymerase activity has been experimentally verified (Hsieh et al., 2017; Majkowska-Skrobek et al., 2018). ^*b*^RBP with a lower probability on depolymerizing activity. ^*c*^RBP without enzymatic activity. ^*d*^The N-terminal part of this protein is lacking under the corresponding accession code, yet the full protein is encoded by nucleotide positions 38449–40114 and 1–1388 of the full genome (NC_028688.1). BLASTp was used as computational alignment algorithm and pairwise alignments were performed against the corresponding first and second RBP from phage KP32, respectively. The accession number of each RBP is given, along with its length and alignment characteristics (cover-coverage, E-value,% identity, identity range-number of identical amino acids/length) of the region over which identical amino acids are found by Blastp, starting from the N-terminus (amino acid 1). HHPred analysis revealed homology in the first RBPs to T4gp10-like domains: Group A – 197–256 aa compared to 170–246 aa of T4gp10 (Probability 86.48) and 206–254 aa compared to 299–383 aa of T4gp10 (Probability 82.04); Group C – 188–256 aa compared to 163–246 aa of T4gp10 (Probability 85.35) and 209–254 aa compared to 303–383 aa of T4gp10 (Probability 84.24); Group D – 196–257 aa compared to 169–246 aa of T4gp10 (Probability 60.26) and 204–255 aa compared to 298–383 aa of T4gp10 (Probability 71.57). Values are shown for KP32 (group A), KpV767 (group C) and 2044-307w (group D). In group B no homology to T4gp10-like domain was detected.*

The second RBP that interrupts the gene synteny in group A KP32viruses is recently demonstrated to have depolymerase activity against capsular serotype K21, whereas the first RBP has depolymerase activity against capsular serotype K3 (Majkowska-Skrobek et al., 2018). These specificities correspond to the host spectrum of phage KP32. Other phages of group A KP32viruses also possess this second putative RBP. The second RBP has no conserved N-terminal anchor domain but has a peptide sequence that is conserved across group A KP32viruses with a consensus sequence over the first 29 amino acids (Supplementary Figure S1B). Similarly to the phage G7C RBP system this conserved peptide may be responsible for attachment to the T4gp10-like domain present in the first RBP. Also for the second RBPs, there is a high diversity in the central sequence with a few exceptions. E.g., in phage K5 and KP32, a highly similar sequence is observed, which hints that the second RBP of phage K5 also targets capsular serotype K21. No chaperone is predicted in any second RBP. Integrating these elements, we model the structural organization of group A KP32viruses as depicted in Figure 2B with a conserved anchor-branched attachment mode but with swapped enzymatic domains for specific capsule/host recognition.

Group B KP32viruses (Table 1) have a simpler RBP organization with a single anchor-based RBP. Six out of seven analyzed phages have an RBP with a putative enzymatic domain, while the seventh phage (IME321) apparently lacks enzymatic activity and might rather encode a tail fiber. The N-terminal conserved anchor domain is shorter (166 amino acids) compared to the corresponding domain in group A KP32viruses (307 amino acids). The RBP also lacks a T4gp10-like domain, which is consistent with the absence of a second RBP in group B KP32viruses (Figure 2).

Phage KpV767 (Table 1) represents another variant of KP32viruses (coined group C). The phage has a first anchor-based RBP, including a fragment of a T4gp10-like domain, but the second RBP is largely truncated to only 69 amino acids, including the conserved N-terminal 29 amino acids for attachment to the T4gp10-like domain (Supplementary Figure S1B). KpV767 appears to result from a retrograde evolution, having lost the potential to infect hosts belonging to two different serotypes.

Finally, phage 2044-307w (group D) is as an opposite example of truncation. The first RBP lacks an enzymatic or receptor binding domain but contains an N-terminal anchor including a fragment of a T4gp10-like domain, while the second tail fiber is a full-featured RBP that contains a conserved N-terminal peptide and a depolymerase domain (Supplementary Figure S1B).

#### KP34viruses

**FIGURE 3:**
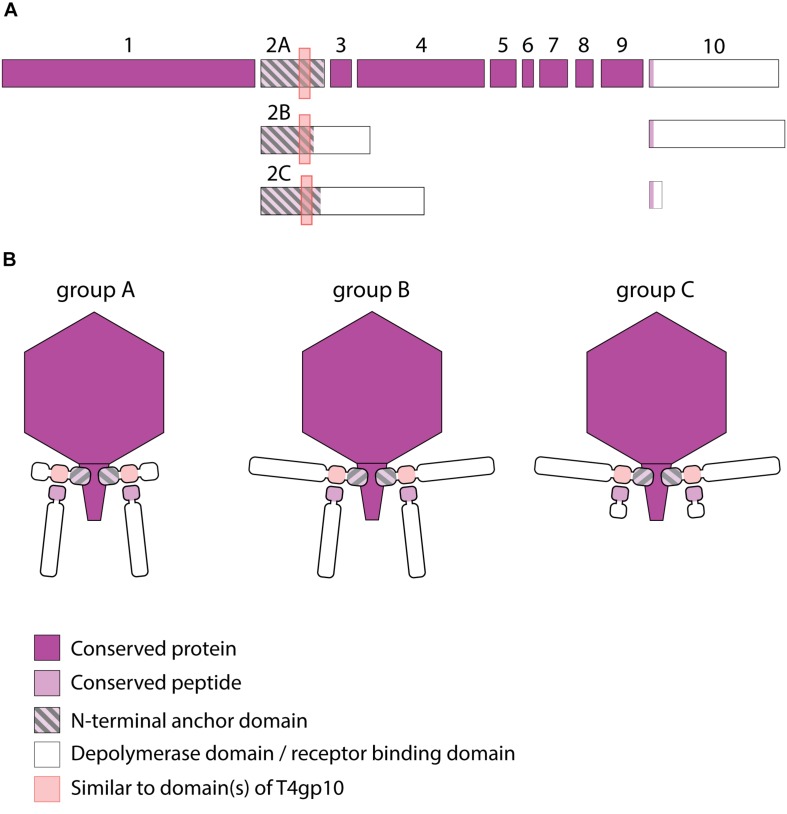
Receptor binding protein systems of KP34viruses. Phages and their RBPs that are proposed to follow these systems, including their grouping into groups A, B and C, are summarized in Table 2. **(A)** The modular composition of RBP genes of phages belonging to three different groups (A, B, and C) is shown relative to the broken gene synteny of phage KP34. For simplicity, only one conserved gene preceding the first RBP and the conserved, intervening genes of phage KP34 are shown. Annotations are given according to GenBank or according to their modeled function in this study (between brackets): (1) – putative internal core protein; (2) – putative tail fiber protein (2A – anchor; 2B,C – anchor with depolymerase); (3) – putative DNA maturase A; (4) – putative DNA maturase B; (5) – hypothetical protein; (6) – hypothetical protein; (7) – hypothetical protein; (8) – hypothetical protein; (9) – endolysin; (10) – hypothetical protein (depolymerase with conserved peptide). **(B)** Schematic models of RBP systems in phage particles of KP34viruses. Group A – two RBPs: anchor-branch attachment mode, first RBP truncated; Group B – two RBPs: anchor-branch attachment mode; Group C – two RBPs: anchor-branch attachment mode, second RBP truncated.

Seventeen phages from the genus of KP34viruses were analyzed (Table 2). Potential proteins involved in host cell recognition could be clearly identified as two genes interrupting the synteny of highly conserved structural genes and genes required for phage particle maturation. Interestingly, both genes are not clustered as in KP32viruses, but are separated by five to eight intervening genes encoding DNA maturases, hypothetical proteins and endolysins, depending on the specific phage. Three different groups (A, B, and C) can be categorized based on differences in length of both genes. Ten group A phages have a short first protein of approximately 300 amino acids annotated as tail fiber. This protein does not encode a putative enzymatic domain, but its N-terminal domain shows high homology to the N-terminus of the phage T7 tail fiber (pfam03906, aa 14–142), similar to the first RBPs of KP32viruses. In addition, the protein contains a fragment of a T4gp10-like domain located at its C-terminus (aa 186–242), which may serve as the attachment point for the second RBP. This protein is highly conserved among all phages of group A KP34viruses (at least 74% identity) (Supplementary Figure S1C). The second RBP sequence encodes a putative enzymatic domain with most such domains forming a β-helical structure. The N-terminal heptapeptide of these proteins contains universally conserved hydrophobic residues (MALxxLV) (Supplementary Figure S1D). These observations suggest that the organization of the RBP apparatus of group A KP34viruses is similar to the system of phage 2044-307w (group D KP32viruses), albeit with a much shorter conserved peptide (Figure 3). Similar short conserved peptides (heptapeptide and undecapeptide) for interaction with the anchor protein have been observed for *E. coli* phages K1E and K1-5 and *Salmonella* phage SP6 (Leiman et al., 2007).

**TABLE 2:** Overview of RBPs of KP34viruses with (predicted) depolymerase activity grouped according to the different observed RBP systems.

**Phage**	**First RBP (protein 2,** Figure 3)						**Second RBP (protein 10,** Figure 3)		
	**Accession number**	**Number of aa**	**Alignment with KP34 first RBP**				**Accession number**	**Number of aa**	**Alignment with KP34 second RBP**
			**Cover**	**E-value**	**Identity**	**Identity range**			
**Group A (two RBPs: anchor-branch attachment mode, first RBP truncated)**								
KP34	YP_003347643.1^c^	307 aa	100%	0.0	100%	307/307	YP_003347651.1	630 aa	
SU503	YP_009199929.1^c^	307 aa	100%	0.0	94%	288/307	YP_009199937.1	500 aa	
F19	YP_009006065.2^c^	307 aa	100%	0.0	93%	284/307	YP_009006074.1	577 aa	
KpV475	YP_009280712.1^c^	318 aa	100%	0.0	88%	279/318	YP_009280720.1^b^	651 aa	
KpV71	YP_009302749.1^c^	318 aa	100%	0.0	91%	288/318	YP_009302756.1^a^	651 aa	
NTUH-K2044-K1-1	YP_009098379.1^c^	318 aa	100%	0.0	90%	287/318	YP_009098385.1^a^	651 aa	No similarity except for the short conserved heptapeptide
KPV811	APD20665.1^c^	318 aa	100%	0.0	87%	278/318	APD20657.1	563 aa	
KpV48	AOZ65257.1^c^	318 aa	100%	0.0	86%	275/318	AOZ65265.1	669 aa	
phiBO1E	AIT13620.1^c^	318 aa	100%	0.0	86%	275/318	AIT13628.1	494 aa	
AltoGao	ASV44938.1^c^	307 aa	99%	2E-162	74%	227/305	ASV44946.1	563 aa	
myPSH1235	30838–31279^c,d^	314 aa	99%	2E-172	76%	239/316	35768–37564^d^	598 aa	
**Group B (two RBPs: anchor-branch attachment mode)**									
Kp2	YP_009188359.1^c^	530 aa	83%	6E-137	81%	207/256	YP_009188367.1	660 aa	
SU552A	YP_009204835.1	793 aa	81%	3E-111	65%	170/261	YP_009204843.1	548 aa	
KpV41	YP_009188788.1^b^	858 aa	94%	2E-110	57%	187/327	YP_009188797.1^b^	651 aa	
phiKpS2	AWK24039.1	946 aa	85%	1E-107	63%	174/275	AWK24047.1	581 aa	
KpV74	APZ82760.1^c^	602 aa	84%	1E-129	72%	186/260	APZ82768.1^a^	577 aa	
**Group C (two RBPs: anchor-branch attachment mode, second RBP truncated)**									
KP-Rio/2015	36399–38783 ^d^	794 aa	90%	9E-116	60%	175/290	42918–43105^c,d^	61 aa	

*The different RBP systems of KP34viruses are visualized in Figure 3. ^*a*^RBP for which the depolymerase activity has been experimentally verified (Lin et al., 2014; Solovieva et al., 2018). ^*b*^RBP with a lower probability on depolymerizing activity. ^*c*^RBP without enzymatic activity. ^*d*^Protein is not annotated in the genome, but the nucleotide positions of the open reading frame are indicated instead. BLASTp was used as computational alignment algorithm and pairwise alignments were performed against the corresponding first RBP from phage KP34. The accession number of each RBP is given, along with its length and alignment characteristics (cover-coverage, E-value,% identity, identity range-number of identical amino acids/length) of the region over which identical amino acids are found by Blastp, starting from the N-terminus (amino acid 1). HHPred analysis revealed homology in first RBPs to T4gp10-like domains: Group A – 186–242 aa compared to 165–244 aa of T4gp10 (Probability 82.81) and 200–243 aa compared to 303–384 aa of T4gp10 (Probability 94.36); Group B – 187–242 aa compared to 167–244 aa of T4gp10 (Probability 83.96) and 200–243 aa compared to 303–384 aa of T4gp10 (Probability 94.63); Group C – 196–253 aa compared to 165–245 aa of T4gp10 (Probability 79) and 210–253 aa compared to 303–384 aa of T4gp10 (Probability 91.72). Values are shown for KP34 (group A), Kp2 (group B), and KP-Rio/2015 (group C).*

Group B KP34viruses contain large first RBPs with a size between 530 and 948 aa. Four out of five RBPs encode an enzymatic domain in the C-terminal or central part of the protein. The corresponding gene in the fifth virus (phage KpV74) contains no predicted enzymatic domain. Group B KP34viruses also encode a second RBP with a predicted enzymatic activity and the same conserved heptapeptide motif as in the second RBP of group A KP34viruses (MALxxLV). The organization of the RBPs in group B KP34viruses is thus similar to that of group A KP32viruses. We found two incongruences in this genus, specifically viruses KP-Rio/2015 and myPSH1235. They both share the gene synteny of KP34viruses but no RBPs were annotated in their genomes. Further genome analysis revealed two open reading frames that presumably fulfill the role of RBPs. We found that phage myPSH1235 follows the RBP organization of group A KP34viruses, while phage KP-Rio/2015 encodes a large first RBP with a predicted enzymatic activity (and a fragment of a T4gp10-like domain) and a second protein that is only 61 aa long, which likely represents a truncated, non-functional RBP. Therefore, phage KP-Rio/2015 forms a different group C with an RBP organization analogous to KP32viruses group C.

### RBPs From Selected *Klebsiella* Myoviruses

Myoviruses have a contractile tail with a baseplate at the head-distal end of the tail. The tail fibers are directly connected to this baseplate. In addition, there is often a central spike (sometimes annotated as ‘fiber’) protruding from the baseplate. Nine *Klebsiella* phages analyzed in this study belong to three different myovirus groups (JD001 group, Menlow group, and ΦK64-1 group) with the latter two groups having a potentially broad host spectrum since they encode between five and nine (Menlow group; phage RaK2) (Hsu et al., 2013; Simoliūnas et al., 2013) or even 11 different depolymerases (phage ΦK64-1) (Pan et al., 2017) (Supplementary Tables S1C–E), necessitating elaborated structural organizations for RBP attachment. We should note that the JD001, Menlow, and ΦK64-1 phages are no taxonomic groups but were grouped in this study for their genetic similarities in the RBP genes. In addition, viruses belonging to the Menlow group have been recently reclassified from *Myoviridae* to *Ackermannviridae* (Adriaenssens et al., 2018). *Ackermannviridae* are characterized by a conserved genome organization and have typical morphology of myoviruses (long contracting tail) but with a different distal end of the tail, which ends with “stars” or “prongs,” being identified as tailspikes (Day et al., 2018).

#### Viruses Belonging to the JD001 Group

**FIGURE 4:**
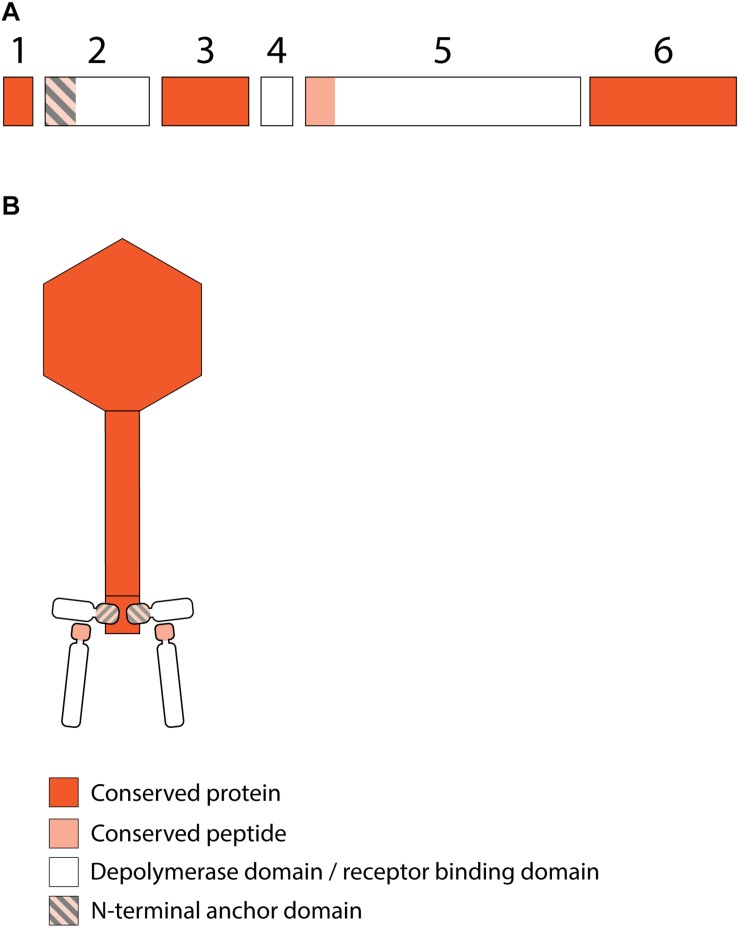
Receptor binding protein systems of viruses of the JD001 group. Phages and their RBPs that are proposed to follow this system are summarized in Table 3. **(A)** The modular composition of RBP genes is shown relative to the broken gene synteny of JD001. Two flanking conserved hypothetical genes are shown. Annotations are given according to GenBank or according to their modeled function in this study (between brackets): (1) – hypothetical protein; (2) – putative tail fiber protein (anchor); (3) – hypothetical protein; (4) – hypothetical protein; (5) – gluconolaconase (depolymerase with conserved peptide); (6) – hypothetical protein. **(B)** Schematic model of the RBP system in JD001 group with an anchor-branch attachment mode and the first RBP truncated.

The putative RBP genes of the viruses of the JD001 group (Table 3) were identified in a region of hypothetical proteins, preceding the DNA polymerase gene. Both genes are located at separate sites with two (JD001, KpV52) or three (KpV79) intervening genes. They all encode a single putative depolymerase, annotated as gluconolaconase, putative tail fiber family protein or tail fiber protein/pectate lyase superfamily protein, respectively. This RBP with depolymerase activity is most likely attached to the anchor protein via a conserved N-terminal domain of about 70 aa, which is distinct from the conserved peptides/domains found in both KP32- and KP34viruses. The anchor protein has no T4gp10-like domain, indicating a different mechanism of interaction (Figures 4A,B).

**TABLE 3:** Overview of RBPs of phages belonging to the JD001 group and with (predicted) depolymerase activity.

			**Alignment with JD001 protein**			
**Phage**	**Accession number**	**Number of aa**	**Coverage**	**E-value**	**Identity**	**Identity range**
**First RBP with anchor truncated (protein 2,** Figure 4)						
JD001	YP_007392884.1^c^	285 aa	100%	0.0	100%	285/285
KpV52	AOZ65389.1^c^	297 aa	38%	5E-53	83%	91/109
KpV79	ATI16499.1^c^	199 aa	29%	3E-46	77%	64/83
**Second RBP with conserved peptide (protein 5,** Figure 4)						
JD001	YP_007392887.1	757 aa	100%	0.0	100%	757/757
KpV52	AOZ65386.1	668 aa	25%	1E-16	51%	85/166
KpV79	ATI16495.1	721 aa	100%	2E-115	37%	289/780

*The RBP system of phages belonging to the JD001 group is visualized in Figure 4. ^*c*^RBP without enzymatic activity. BLASTp was used as computational alignment algorithm and pairwise alignments were performed against the corresponding first and second RBP from phage JD001, respectively. The accession number of each RBP is given, along with its length and alignment characteristics (cover-coverage, E-value,% identity, identity range-number of identical amino acids/length) of the region over which identical amino acids are found by Blastp, starting from the N-terminus (amino acid 1).*

#### Viruses Belonging to the Menlow Group

**FIGURE 5:**
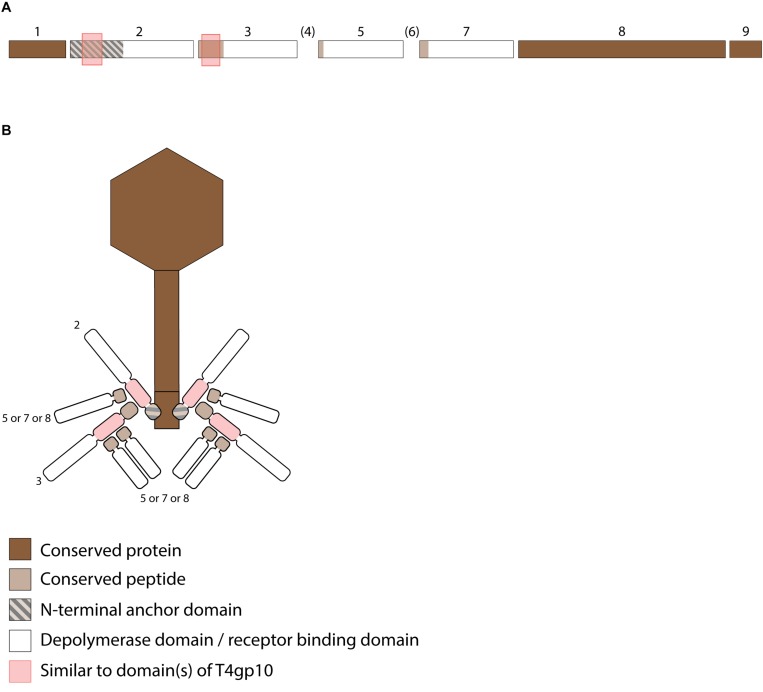
Receptor binding protein systems of the Menlow group. Phages and their RBPs that are proposed to follow this system are summarized in Table 4. **(A)** The modular composition of the RBP genes is shown relative to the broken gene synteny of Menlow. Annotations are given according to GenBank or according to their modeled function as annotated in this study (between brackets): (1) – putative tail protein; (2) – tail spike protein (anchor with depolymerase); (3) – tail spike protein (depolymerase with conserved peptide); (4) – hypothetical protein; this protein is not present in all Menlow group phages; (5) – tail spike protein (depolymerase with conserved peptide); (6) – hypothetical protein; this protein is not present in all Menlow group phages; (7) – tail spike protein (depolymerase with conserved peptide); (8) – hypothetical protein (depolymerase); (9) – neck protein. **(B)** Schematic model of the RBP system in Menlow with an anchor-mulibranched attachment mode.

The viruses of the Menlow group encode, amid a conserved synteny of structural genes, four non-conserved putative RBPs and one conserved RBP, all with putative depolymerase activity (Figure 5A). Phages KpS110 and 0507-KN2-1 encode an additional sixth RBP with a predicted depolymerase domain (Table 4). The first two non-conserved RBPs (protein 2 and 3 in Figure 5) have N-terminal domains of 412 and 195 aa long, respectively, which is conserved among the four members of the Menlow group. The following two non-conserved RBPs have a shorter domain/peptide of 38 and 67 aa, respectively, conserved among all members of the Menlow group (Supplementary Figures S1E–H).

**TABLE 4:** Overview of RBPs of phages belonging to the Menlow group and with (predicted) depolymerase activity.

			**Alignment with Menlow RBP**			
**Phage**	**Accession number**	**Number of aa**	**Coverage**	**E-value**	**Identity**	**Identity range**
**First RBP with anchor (protein 2,** Figure 5)						
Menlow	AUG87748.1	960 aa	100%	0.0	100%	960/960
KpS110	AUV59228.1^c^	960 aa	42%	0.0	95%	383/424
May	AUG87958.1	1180 aa	44%	0.0	90%	392/412
0507-KN2-1	YP_008532046.1^c^	1072 aa	43%	0.0	90%	385/427
**Second RBP with conserved peptide (protein 3,** Figure 5)						
Menlow	AUG87749.1	768 aa	100%	0.0	100%	768/768
KpS110	AUV59230.1	857 aa	25%	4E-120	96%	189/196
May	AUG87959.1^b^	1288 aa	27%	1E-105	84%	178/212
0507-KN2-1	YP_008532047.1^a^	1245 aa	25%	5E-112	92%	180/195
**Third RBP with conserved peptide (protein 5,** Figure 5)						
Menlow	AUG87751.1^b^	660 aa	100%	0.0	100%	660/660
KpS110	AUV59232.1	674 aa	13%	6E-22	60%	55/91
May	AUG87960.1^b^	660 aa	100%	0.0	99%	657/660
0507-KN2-1	YP_008532048.1^b^	657 aa	27%	8E-37	48%	91/190
**Fourth RBP with conserved peptide (protein 7,** Figure 5)						
Menlow	AUG87753.1	730 aa	100%	0.0	100%	730/730
KpS110	AUV59234.1	685 aa	19%	1E-40	61%	86/141
May	AUG87962.1	692 aa	16%	2E-40	71%	86/121
0507-KN2-1	YP_008532049.1^b^	607 aa	14%	1E-35	72%	79/109
**Fifth RBP (protein 8,** Figure 5)						
Menlow	AUG87754.1^b^	1612 aa	100%	0.0	100%	1612/1612
KpS110	AUV59236.1^b^	1612 aa	100%	0.0	99%	1602/1612
May	AUG87963.1^b^	1612 aa	100%	0.0	99%	1591/1612
0507-KN2-1	YP_008532051.1^b^	1616 aa	100%	0.0	99%	1604/1612
**Additional RBP (not present in genome of Menlow)**						
Menlow	No protein					
KpS110	AUV59229.1	554 aa	Not applicable			
May	No protein					
0507-KN2-1	YP_008532050.1	542 aa				

*The RBP system of phages belonging to the Menlow group is visualized in Figure 5. ^*a*^RBP for which the depolymerase activity has been experimentally verified (Hsu et al., 2013). ^*b*^RBP with a lower probability on depolymerizing activity. ^*c*^RBP without enzymatic activity. BLASTp was used as computational alignment algorithm and pairwise alignments were performed against the respective RBP from phage Menlow. The accession number of each RBP is given, along with its length and alignment characteristics (cover-coverage, E-value,% identity, identity range-number of identical amino acids/length) of the region over which identical amino acids are found by Blastp, starting from the N-terminus (amino acid 1). HHPred analysis revealed homology in RBPs to T4gp10-like domains: First RBP (protein 2) – 94–252 aa compared to 61–248 aa of T4gp10 (Probability 99.88) and 92–251 aa compared to 214–386 aa of T4gp10 (Probability 44.31); Second RBP (protein 3) – 26–204 aa compared to 68–296 aa of T4gp10 (Probability 94.08) and 26–162 aa compared to 165–384 aa of T4gp10 (Probability 95.39) and 41–83 aa compared to 303–383 aa of T4gp10 (Probability 73.93). Values are shown for template phage Menlow.*

To explore how this high number of putative RBPs might be structurally organized, we searched for homology to T4gp10-like domains 2/3 and N-terminally conserved domains/peptides as they suggest branching points. Two domains homologous to T4gp10 were located in the N-terminal part of RBP 2 (RBP with anchor domain) and RBP 3, whereas RBPs 3, 5, 7 (Figure 5A) contain conserved peptides in their N-terminus. A fifth RBP (protein 8 is present and highly identical) in all members of the Menlow group, while a sixth RBP with putative depolymerase activity is only present in phage KpS110 and phage 0507-KN2-1. Integrating the presence/absence of these structural elements (Figure 5B) a possible model implies that the first RBP (protein 2, Figure 5A) is directly attached to the tail *via* a conserved N-terminal anchor and that its T4gp10-like domain probably provides an attachment site for at least two RBPs (3 and 5 or 7 or 8, Figure 5B). Subsequently, the second RBP (protein 3) provides attachment sites *via* its T4gp10-like domains for two more RBPs (proteins 5 or 7 or 8). Together they constitute a unit of branched tail fibers. The highly conserved fifth RBP (protein 8) may be the central tail fiber that protrudes from below the plane of the baseplate (Nobrega et al., 2018). More structural and genetic studies will be needed for an improved understanding of the elaborated RBP system in viruses from the Menlow group.

#### Viruses Belonging to the ΦK64-1 Group

**FIGURE 6:**
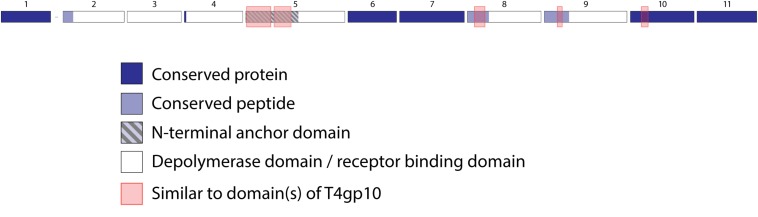
Receptor binding protein systems of phage ΦK64-1 and RaK2. Phages and their RBPs that are proposed to follow this system are summarized in Table 5. The modular composition of RBP genes is shown relative to the broken gene synteny. Annotations are given according to GenBank or according to their modeled function in this study (between brackets): (1) – putative tail fiber protein (depolymerase); (2) – tail spike protein (depolymerase with conserved peptide); (3) – tail spike protein (depolymerase); (4)– putative tail fiber protein (depolymerase with conserved peptide); (5) – putative tail fiber protein (anchor with depolymerase); (6) – putative tail fiber protein (depolymerase); (7) – putative structural protein (depolymerase); (8) – putative tail fiber protein (depolymerase with conserved peptide); (9) – putative tail fiber protein (depolymerase with conserved peptide); (10) – putative structural protein (depolymerase); (11) – putative tail fiber protein (depolymerase).

*Klebsiella* phages belonging to ΦK64-1 group (ΦK61-1 and RaK2; Supplementary Table S1E) have likely evolved the most elaborate RBP apparatus (Table 5). ΦK64-1 encodes 11 proteins recognized as putative depolymerases, while in the genome of RaK2 10 putative depolymerases are predicted. The middle and C-terminal regions of five RBPs are different between the corresponding genes of ΦK61-1 and RaK2, reflecting the diversity of capsular serotypes that can be recognized by putative depolymerases of these two phages, whereas the middle and C-terminal parts of other RBPs show more than 75% identity between both phages, suggesting an overlap in the host spectrum (Pan et al., 2017). We found in this study that these proteins also contain a slew of structural elements found in other complex tail fiber machineries such as one N-terminal anchor domain, four short conserved peptides at the N-terminus and five T4gp10-like domains (Supplementary Figures S1I–M), indicating that phages of the ΦK64-1 group also re-use standardized units to build up a highly complex RBP apparatus (Figure 6).

**TABLE 5:** Overview of RBPs of phages belonging to the ΦK64-1 group and with (predicted) depolymerase activity.

	**RBP**					
			**Alignment with** Φ**K64-1 RBP**			
**Phage**	**Accession number**	**Number of aa**	**Coverage**	**E-value**	**Identity**	**Identity range**
**First RBP (protein 1,** Figure 6)						
ΦK64-1	YP_009153165.1^b^	595 aa	100%	0.0	100%	595/595
RaK2	YP_007007253^b^	595 aa	100%	0.0	100%	595/595
**Second RBP with conserved peptide (protein 2,** Figure 6)						
ΦK64-1	YP_009153195.1^a^	736 aa	100%	0.0	100%	736/736
RaK2	YP_007007681.1^b^	580 aa	15%	3E-30	61%	75/122
**Third RBP (protein 3,** Figure 6)						
ΦK64-1	YP_009153196.1^a^	651 aa	100%	0.0	100%	651/651
RaK2	no protein					
**Fourth RBP with conserved peptide (protein 4,** Figure 6)						
ΦK64-1	YP_009153197.1^a^	702 aa	100%	0.0	100%	702/702
RaK2	YP_007007682.1	715 aa	96%	2E-39	26%	187/718
**Fifth RBP with anchor (protein 5,** Figure 6)						
ΦK64-1	YP_009153198.1^a^	1193 aa	100%	0.0	100%	1193/1193
RaK2	YP_007007683.1	1113 aa	52%	0.0	82%	518/633
**Sixth RBP (protein 6,** Figure 6)						
ΦK64-1	YP_009153199.1^a^	584 aa	100%	0.0	100%	584/584
RaK2	YP_007007684.1	584 aa	100%	0.0	99%	581/584
**Seventh RBP (protein 7,** Figure 6)						
ΦK64-1	YP_009153200.1^a^	779 aa	100%	0.0	100%	779/779
RaK2	YP_007007685.1	779 aa	100%	0.0	97%	754/779
**Eighth RBP with conserved peptide (protein 8,** Figure 6)						
ΦK64-1	YP_009153201.1^a^	888 aa	100%	0.0	100%	888/888
RaK2	YP_007007686.1	895 aa	29%	1E-147	89%	231/259
**Ninth RBP with conserved peptide (protein 9,** Figure 6)						
ΦK64-1	YP_009153202.1^a^	996 aa	100%	0.0	100%	996/996
RaK2	YP_007007687.1	806 aa	28%	2E-129	76%	225/298
**Tenth RBP (protein 10,** Figure 6)						
ΦK64-1	YP_009153203.1^a^	767 aa	100%	0.0	100%	767/767
RaK2	YP_007007688.1	767 aa	100%	0.0	90%	690/767
**Eleventh RBP (protein 11,** Figure 6)						
ΦK64-1	YP_009153204.1^b^	719 aa	100%	0.0	100%	605/605
RaK2	YP_007007689.1^b^	688 aa	100%	0.0	75%	454/605

*The gene syntheny of phages belonging to the ΦK64-1 group is visualized in Figure 6. ^*a*^RBP for which the depolymerase activity has been experimentally verified (Pan et al., 2017). ^*b*^RBP without enzymatic activity. BLASTp was used as computational alignment algorithm and pairwise alignments were performed against the respective RBP from phage ΦK64-1, respectively. The accession number of each RBP is given, along with its length and alignment characteristics (cover-coverage, E-value,% identity, identity range-number of identical amino acids/length) of the region over which identical amino acids are found by Blastp, starting from the N-terminus (amino acid 1). HHPred analysis revealed homology in RBPs to T4gp10-like domains: Fifth RBP (protein 5) – 113–305 aa compared to 68–384 aa of T4gp10 (Probability 93.67) and 108–241 aa compared to 160–385 aa of T4gp10 (Probability 90.92) and 130–171 aa compared to 305–384 aa of T4gp10 (Probability 48.74); Eighth RBP (protein 8) – 67–221 aa compared to 151–393 aa of T4gp10 (Probability 96.28) and 95–138 aa compared to 303–384 aa of T4gp10 (Probability 74.61) and 79–214 aa compared to 66–247 aa of T4gp10 (Probability 71.93); Ninth RBP (protein 9) – 175–225 aa compared to 303–390 aa of T4gp10 (Probability 92.73) and 160–219 aa compared to 164–245 aa of T4gp10 (Probability 90.38); Tenth RBP (protein 10) – 165–217 aa compared to 303–392 aa of T4gp10 (Probability 89.93) and 123–209 aa compared to 136–254 aa of T4gp10 (Probability 89.87). Values are shown for template phage ΦK64-1.*

### RBPs From *Klebsiella* Siphoviruses

#### KP36viruses

**FIGURE 7:**
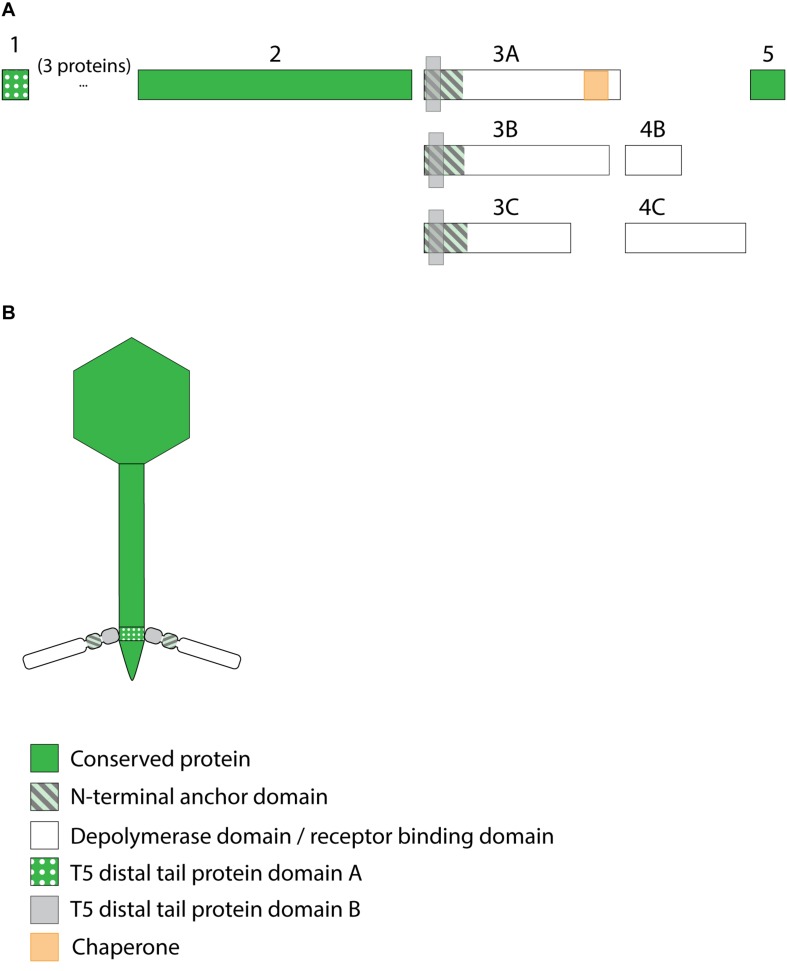
Receptor binding protein systems of the KP36viruses. Phages and their RBPs that are proposed to follow this system, including their grouping into groups A, B and C, are summarized in Table 6. **(A)** The modular composition of the RBP genes is shown relative to the broken gene synteny of the reference phage KP36. Annotations are given according to GenBank or according to their modeled function in this study (between brackets): (1) – minor tail protein; (2) – tail fiber protein; (3) – putative tail fiber protein (3A,B – anchor with depolymerase; 3C – anchor); (4) – hypothetical protein (4B,C – depolymerase); (5) – putative single-stranded DNA binding protein. Proteins 1, 2, and 5 are present in all KP36viruses. **(B)** Schematic model of the RBP system in phage particles of KP36viruses group A with a split T5 distal tail protein. Domain A is encoded by short minor tail protein forming a ring at the end of the phage tail tube and offers an attachment point for domain B, which is incorporated in the anchoring part of the RBP that represents the putative side tail fiber.

All 12 identified *Klebsiella* siphoviruses belong to the KP36viruses. They are also featured by a synteny of genes encoding structural proteins such as the tail length tape-measure protein, minor tail proteins and a putative tail assembly protein. This synteny is disrupted by one or two genes, depending on the phage. Three groups can be categorized with the majority of phages belonging to group A, while phage PKP126 (group B) and phage 1513 (group C) represent exceptions from the general structure of group A (Figure 7A and Table 6). Members of group A KP36viruses (including the reference phage KP36) have a single predicted RBP with putative depolymerase activity. It has been demonstrated that the RBP of KP36 is enzymatically active against capsular serotype K63 (Majkowska-Skrobek et al., 2016). The modular structure of this RBP is similar to that of the RBP of group B KP32viruses, having an N-terminal anchor domain, a highly variable central domain with enzymatic activity, and a C-terminal chaperone. KP36viruses belonging to group B and group C also have an RBP with a similar N-terminal anchor domain (Supplementary Figure S1N). Phage PKP126 RBP (group B) has a predicted enzymatic activity in the central domain in contrast to the truncated RBP of phage 1513 (group C). The chaperone domain is missing in the RBP of both groups B and C.

**TABLE 6:** Overview of RBPs of phages belonging to the KP36viruses and with (predicted) depolymerase activity.

**Phage**	**RBP (protein 3,** Figure 7)					
			**Alignment with KP36 RBP**			
	**Accession number**	**Number of aa**	**Coverage**	**E-value**	**Identity**	**Identity range**
**Group A (RBP with anchor)**						
KP36	YP_009226011.1^a^	883 aa	100%	0.0	100%	883/883
KLPN1	YP_009195383.1	756 aa	30%	3E-99	67%	187/280
KOX1	ARM70347.1	765 aa	21%	1E-89	90%	170/189
JY917	AVI03134.1^c^	812 aa	21%	2E-88	86%	173/201
Sushi	YP_009196676.1^b^	832 aa	18%	2E-75	82%	144/176
MezzoGao	ASV44964.1^b^	973 aa	20%	1E-72	78%	152/194
NJS1	AXF39389.1^b^	992 aa	18%	4E-72	81%	142/176
GML-KpCol1	AUE22051.1^b^	972 aa	18%	8E-71	80%	140/176
KpV522	AOZ65310.1^b^	1141 aa	20%	1E-68	72%	139/193
KPN N141	ASW27458.1^b^	879 aa	18%	1E-59	80%	140/174
**Group B (RBP with anchor plus second depolymerase)**						
PKP126	YP_009284923.1^b^	833 aa	18%	1E-70	76%	137/180
	YP_009284924	251 aa	hypothetical protein, no similarity			
**Group C (RBP with anchor plus second RBP)**						
1513	YP_009197878.1^c^	659 aa	21%	4E-86	88%	171/194
	YP_009197879.1	541 aa	Hypothetical protein, no similarity			

*The RBP system of KP36viruses is visualized in Figure 7. ^*a*^RBP for which the depolymerase activity has been experimentally verified (Majkowska-Skrobek et al., 2016). ^*b*^RBP with a lower probability on depolymerizing activity. ^*c*^RBP without enzymatic activity. BLASTp was used as computational alignment algorithm and pairwise alignments were performed against the respective RBP from phage KP36. The accession number of each RBP is given, along with its length and alignment characteristics (cover-coverage, E-value,% identity, identity range-number of identical amino acids/length) of the region over which identical amino acids are found by Blastp, starting from the N-terminus (amino acid 1).*

No T4gp10-like domain was found in the N-terminal region of KP36gp50 (RBP). Instead, a small domain (residues 4–63) homologous to domain B (92–155 aa) of the distal tail protein (Dit or T5pb9) of siphovirus T5 has been detected. Dit is located in the T5 tail tip at the junction between the tail tube and the ultimate conical structure and is composed of two domains. Domain A forms a hexameric structure and connects to the end of the tail tube, whereas domain B constitutes the attachment site for three L-shaped tail fibers (Flayhan et al., 2014). These L-shaped tail fibers initially bind reversibly to polymannose containing O-antigens (Heller and Braun, 1982). Remarkably, domain A of T5pb9 has not been found in KP36gp50 but is instead present in the KP36 minor tail protein (residues 22–77 corresponding to amino acids 27–85 of T5pb9) that is encoded four genes upstream of KP36gp50. This horizontal transfer event indicates that in KP36viruses the conserved minor tail protein only comprises domain A, which is located at the junction of the tail tube and the conical tip of the tail. Domain A of the minor tail protein is proposed to interact with the RBP *via* its N-terminal domain B. This RBP may thus represent the side tail fibers similar to the L-shaped tail fibers in phage T5. In other words, the distal tail protein has been split into two separate elements in KP36viruses.

Phage PKP126 and 1513 (groups B and C, respectively) have an additional RBP with putative depolymerase activity. Its exact role is difficult to predict and typical elements hinting at a specific structural organization such as a conserved peptide or anchor domain are missing. We hypothesize that those enzymes are not incorporated in the phage particle, but rather are produced as soluble proteins. Upon cell lysis the neighboring cells are sensitized for infection through enzymatic removal of the capsule by the soluble, diffusible depolymerase. This mechanism would be especially beneficial for phages lacking depolymerase activity in the their first RBP (e.g., group C phage 1513).

An additional preceding RBP (Figure 7A; protein 2) is highly conserved across all analyzed KP36viruses, except in phage 1513. The role of this RBP is unclear. One possibility is that it is a second side RBP as observed in some T5viruses (DT57C and DT571) (Golomidova et al., 2016; Nobrega et al., 2018). An alternative possibility is that this protein represents the central tail fiber. Given its ambiguous role and location, this RBP was not included in the model depicted in Figure 7B.

## Discussion

In this work we have performed an extensive *in silico* analysis of the RBPs of *Klebsiella* phages genomes with a focus on RBPs with depolymerase activity. The tripartite relationship between depolymerase specificity, capsular serotype and phage host spectrum has now extensively been demonstrated for *Klebsiella* phages (Hsu et al., 2013; Lin et al., 2014; Majkowska-Skrobek et al., 2016; Hsieh et al., 2017; Pan et al., 2017; Solovieva et al., 2018). Podovirus KP32 possesses two experimentally confirmed depolymerases, which are enzymatically active against capsule serotype K3 and K21, respectively. Correspondingly, all strains infected by phage KP32 have either a K3 or K21 serotype (Majkowska-Skrobek et al., 2018). Podovirus KpV71 infects strains with serotype K1, which perfectly matches the specificity of its experimentally verified depolymerase. However, podovirus KpV74, which has also a single RBP, infects strains with serotype K2 and K13. These observations were explained by the structural similarity of capsule types K2 and K13, which were also found to cross-react with specific antibodies (Pieroni et al., 1994; Pan et al., 2015; Volozhantsev et al., 2016; Solovieva et al., 2018). The more diverse capsular specificity of podoviruses KpV763, KpV766, and KpV289 (Volozhantsev et al., 2016; Solovieva et al., 2018) is now explained in this study by the observed presence of two RBPs (Table 1). Some large jumbo phages such as phage ΦK64-1 produce an elaborated, broad-spectrum RBP apparatus. Phage ΦK64-1 encodes 11 putative RBPs from which nine are confirmed to possess enzymatic activity against 10 serotypes in total (K1, K11, K21, K25, K30/K69, K35, K64, KN4, and KN5). Whereas eight RBPs are active against a single but different serotype, the ninth RBP is active against two capsular serotypes (protein 10, Figure 6; active against K30 and K69) (Pan et al., 2017).

Based on the structural knowledge of RBPs of mainly *E. coli* phages such as T7, K1F, K1-5, G7C and T5, we have identified structurally conserved building blocks to model the RBP apparatus of *Klebsiella* phages. The modularity of RBPs in combination with intensive horizontal transfer of genes or gene domains (Casjens and Molineux, 2012) allows for a maximum re-use of conserved, evolutionary optimized elements. Simultaneously, the possibility to rapidly shift the host spectrum based on an exchange of the depolymerase domain is retained. Indeed, specific RBP domains, sometimes in pair with their cognate chaperone, are present in each RBP system. This is well-illustrated by the high similarity of the experimentally verified depolymerase domains of KP36gp50 and KP34gp57. Both proteins target capsular serotype K63, but have either an N-terminal anchor or conserved peptide, respectively.

The high adaptability of *Klebsiella* phage RBPs is essential since *K. pneumoniae* is featured by a high capsular diversity. Consequently, *Klebsiella* phages have often a very narrow spectrum limited to strains from one or two capsular serotypes. Colonization of new niches occupied by *K. pneumoniae* isolates with a different capsular serotype thus necessitates a flexible system for rapid adaptation. In addition, the same flexibility is needed to respond to phenotypic serotype switches of *K. pneumoniae* strains (Pan et al., 2015; Wyres et al., 2015).

**FIGURE 8:**
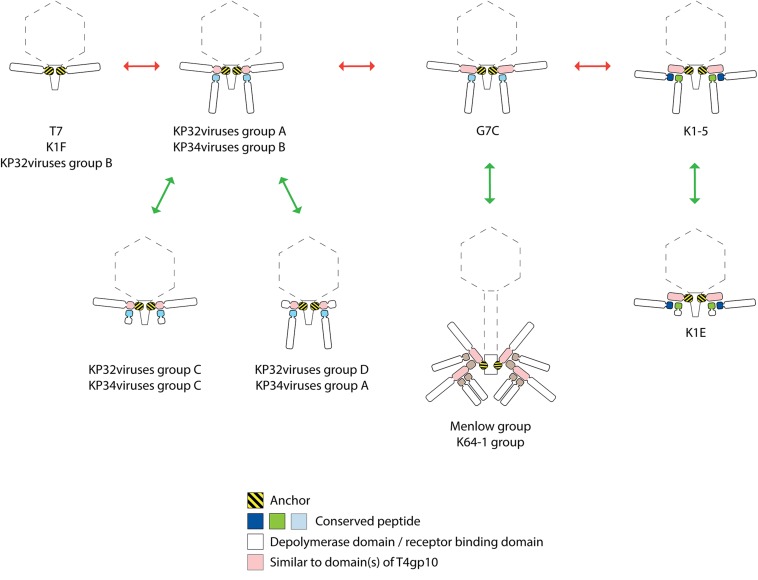
Possible evolutionary linkages between diverse RBP systems, driven by the acquisition and loss of domains. The model RBP systems of phage T7, K1F, G7C, K1-5, and K1-E have been reported before. The RBP systems of KP32viruses groups A, B, C, D (Figure 2 and Table 1), KP34viruses groups A, B, C (Figure 3 and Table 2), phages belonging to the Menlow group (Figure 5 and Table 4) and phage belonging to the ΦK64-1 group (Figure 6 and Table 5) have been studied in this work.

In this study, we propose that RBPs of *Klebsiella* phages are organized according to several distinct systems (Figure 8). The simplest mechanism is similar to the anchor-based system described for phage T7 and K1F. In phages from KP32viruses group B and KP36viruses, the single RBP is directly connected with the phage particle *via* its conserved N-terminal anchor domain. Other phages (KP32viruses group A; KP34 viruses group B) that produce two RBPs encode the structural elements for an anchor-branched mechanism as reported for phage G7C. Here, the first RBP contains a conserved N-terminal anchor serving for attachment to the virion, followed by a specific fragment of a T4gp10-like domain providing the docking site for a second RBP. Notably, the fragment encoding the T4gp10-like docking site in those *Klebsiella* phages is shorter compared to the corresponding domain in T4 and may therefore correspond to a single attachment site. The second RBP is presumably attached *via* a conserved peptide (KP32viruses, KP34viruses, JD001 group, Menlow group, ΦK64-1 group). This conserved peptide is different for each group of phages, varies in length and can be as short as seven amino acids. Such attachment *via* a short peptide is in line with the RBP complex of K1E/K1-5/SP6-like phages where both RBPs carry either a 7- or 11-residue conserved peptide at their respective N-terminus. In the case of *E. coli* phage G7C the shorter G7Cgp63.1 RBP carries a positively charged surface that binds to the T4gp10-like domain of G7Cgp66, yet, the conserved peptides in *Klebsiella* phages lack this positive charge, inferring that different interacting forces take place between the first and second RBP. Similar to the RBPs of phage K1-5, the two experimentally verified depolymerases of phage KP32 target two different capsular serotypes. In both cases the double RBP system thus expands the host spectrum.

The presence of either an anchor- or an anchor-branched system is not directly linked to the taxonomic organization. In addition, there is no sequence homology between functionally similar, structural building blocks across those phage groups. E.g., the first 140 amino acids of the N-terminal anchor domains of RBPs encoded by *Klebsiella* phages belonging to *Podoviridae* show similarity with the well-characterized N-terminal domain of T7 tail fiber and their first 300 amino acids are conserved across different podoviruses analyzed in this study. The T7 tail fiber attaches with its N-terminal anchor domain to the region where the adaptor (gp11) interacts with nozzle (gp12) of the short tail complex (Cuervo et al., 2013). The corresponding proteins of phage KP32 share 62 and 61% sequence identity with the adaptor and nozzle protein of phage T7, respectively, while in the case of KP34 the identity is lower (29% identity with a coverage of 67% for the adaptor protein and 23% identity with a coverage of 98% for the nozzle protein). There is no amino acid similarity between the conserved N-terminal anchor domains in RBPs from different taxonomic groups of *Klebsiella* phages indicating that also the interacting partner in the tail structure has also evolved accordingly. In the case of KP36viruses (*Siphoviridae*), a remarkable horizontal transfer event has taken place between the distal tail protein and the tail fiber of KP36viruses when comparing to the siphovirus T5 model. Domain B of the distal tail protein has been transferred to the N-terminus of the tail fiber protein in KP36viruses. Whereas in phage T5 protein–protein interaction occurs between the N-terminus of the RBP and domain B of the distal tail protein, novel interactions between domain A of the minor tail protein and domain B embedded in the tail fiber must have been evolved to compensate for the loss of interaction by a direct covalent bond as in phage T5.

Interestingly, several phages with a single enzymatic RBP do not follow the anchor system as described for phage T7, but use the anchor-branched system of G7C with either the first (KP32virus group D; KP34virus group A; JD001 group) or second RBP (KP32virus group C; KP34virus group C) being truncated. In the case of KP34viruses it is even the predominant RBP system. The occurrence of these intermediate RBP systems suggests evolutionary linkages between the different RBP architectures. Starting from the simplest organization with a single RBP (T7, K1F, and KP32viruses group B), the acquisition of a fragment of a T4gp10-like domain allowed for the attachment of a second RBP (KP32viruses group A and KP34viruses group B). The first RBP from *E. coli* phage G7C has acquired a full T4gp10-like domain (similarity to both subdomain D2 and D3 of T4gp10), offering a potential second attachment site for a different RBP. This second site is not occupied in phage G7C, whereas the *E. coli* model phages K1-5, SP6, and K1E have effectively two RBPs attached to the same intermediate protein that also comprises both subdomain D2 and D3. In K1-5, SP6 and K1E, this intermediate protein with the full T4gp10-like domain has lost its C-terminal receptor-binding domain, resulting in an ‘adapter’ system – a short protein with two sites for binding two different RBPs and no domain beyond these two domains (Figure 8). It should be noted that a simple adapter protein that provides attachment sites for two RBPs as described for *E. coli* phage K1-5 and *Salmonella* phage SP6 is not observed in the case of *Klebsiella* podoviruses. Obviously, an opposite evolutionary trajectory of RBP systems (from adapter to anchor) cannot be excluded as well. The success of the modular build-up of the RBP apparatus and the extensive number of horizontal transfer events have obscured possible insight in the direction of this evolution. The assumption that evolution generally takes place from simple to more complex systems, hints at the first direction (from anchor to adapter). KP32viruses group C and D, and KP34viruses groups A and C may have lost a second intact RBP by retrograde evolution when thriving in a new environment that is dominated by a single serotype *Klebsiella* strain. Having a truncated second RBP may provide a fitness advantage in such a situation. The truncated RBP may remain as a temporal docking site to acquire a new RBP for host range expansion by horizontal transfer when moving to a niche with different *Klebsiella* serotypes. Phages belonging to the Menlow group and ΦK64-1 group carry multiple RBPs that obviously recycle established structural elements such as a conserved N-terminal domain, short conserved peptides and a T4gp10-like domain (or fragments thereof). Yet, more experimental (structural, genetic, biochemical) studies are required to make a plausible prediction on the structural organization of these elaborated RBP systems.

In summary, we have modeled the organization of diverse RBP systems in *Klebsiella* phages. The modular composition and re-use of established structural domains for anchoring and branching provide the phages the full potential to rapidly shift capsular serotype specificity or to expand the spectrum. We expect that the increasing amount of (meta)genome sequencing data will reveal further evolutionary relationships between some of the groups we describe in this analysis, but the main groups will remain in place. The data available to us today clearly show that the architecture of RBP systems is dominated by horizontal transfer events of modules that can be as small as short peptides to as large as multiple domains. Although our analysis was based on experimentally confirmed interactions of *E. coli* phage RBPs (Leiman et al., 2007; Prokhorov et al., 2017), further experimental validation of the presented models is needed and has already been initiated by our team. To analyze the interactions between the T4gp10-like domains and conserved peptides, protein–protein interactions can be studied by various techniques such as isothermal calorimetry (ITC), two-hybrid systems, surface plasmon resonance (SPR), cryoEM, or an enzyme-linked immunosorbent assay (ELISA). This work also adds improved functional annotations to genes to which previously no specific function has been assigned, but which are putative tail fibers/spikes with depolymerizing activity. The high number of newly predicted depolymerases in this study can be verified by their recombinant production followed by activity tests against strains with particular capsular serotypes.

## Data Availability Statement

The datasets analyzed for this study can be found in the GenBank. All genome and protein accession numbers are listed in the tables and Supplementary Material.

## Author Contributions

AL, ZD-K, and YB designed the *in silico* search approach of RBPs domains and modeled the RBP assembly systems. PL introduced the concept of branching RBPs. AL performed *in silico* analysis and collected the data. AL, PL, ZD-K, and YB analyzed the data and worked on the manuscript. All authors read and accepted the final version of the manuscript.

## Conflict of Interest

The authors declare that the research was conducted in the absence of any commercial or financial relationships that could be construed as a potential conflict of interest.

## Footnotes

1 blast.ncbi.nlm.nih.gov

2 sbg.bio.ic.ac.uk

3 swissmodel.expasy.org

4 hmmer.org

5 toolkit.tuebingen.mpg.de

6 weblogo.berkeley.edu
