# Supplementary material for: Modeling the Architecture of Depolymerase-Containing Receptor Binding Proteins in Klebsiella Phages
Source: Front Microbiol. 2019 Nov 15;10:2649. doi: 10.3389/fmicb.2019.02649 (PMC6872550; doi:10.3389/fmicb.2019.02649)
Supplement: Supplementary file 1 [file Image_1.pdf]

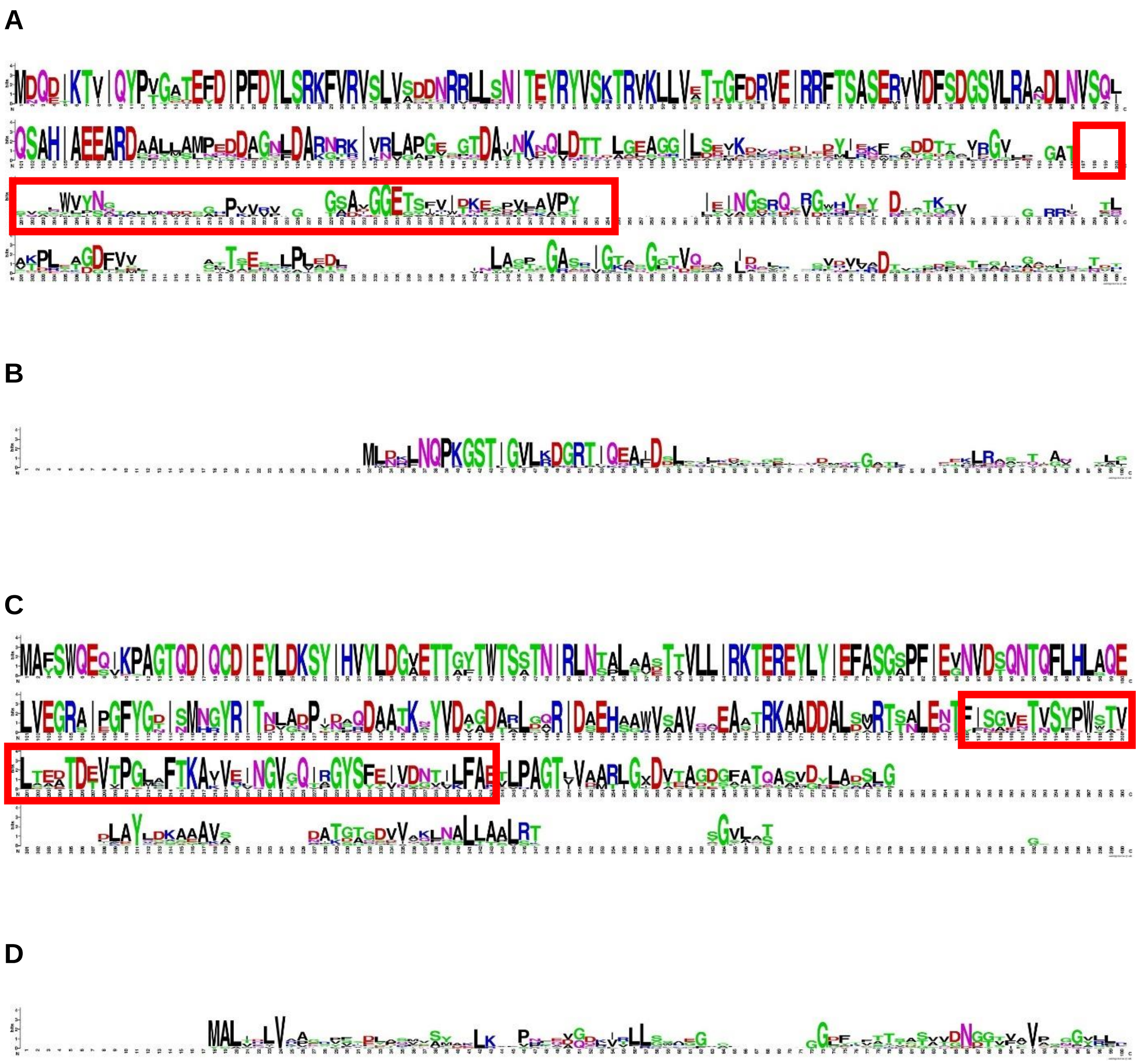

E

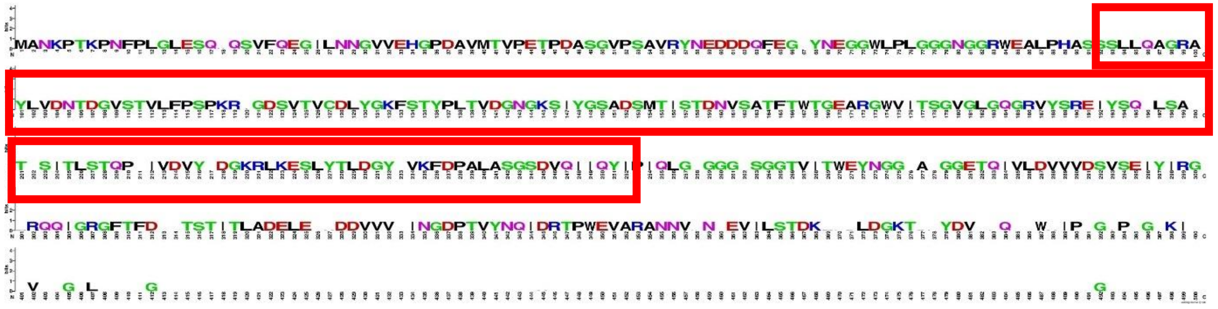

F

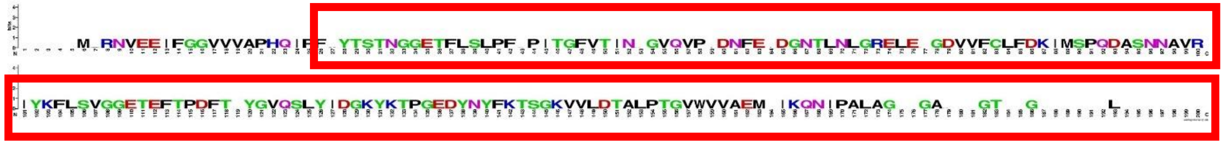

G

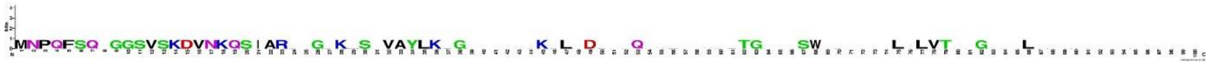

H

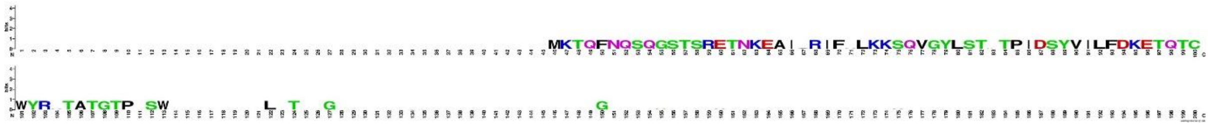

I

```
1 YP_009153195.1 100.0% 100.0%   MTNSLIQPKGSVSKETNIQSIARITGSKIEEVKYLEDGLDIAGLKFVYDSSTETIWKLNGNETGMVDSWNIVDESTIIIV
2 YP_007007681.1  72.7%  20.3%   MSNNLIQPKGSVSKETNIQSIARINGVKISEVKYLEDGLDVTGLKYLYDSSTETVWKLNGDETGLIDDWFIQDETMTI-Q
  consensus/100%                MoNsLIQPKGSVSKETNIQSIARIsGsKIpEVKYLEDGLDlsGLKalYDSSTETlWKLNGsETGhlDsW.I.DEohhI..
```

J

```
1 YP_009153197.1 100.0% 100.0%   MANKLTQPKGSISKETNKEAIARLFGIKKTAVGYISTSVLIDPYTILYDESTET-CWYRGTATGTPISWIITNGSLTLQT
2 YP_007007682.1  95.0%  23.7%   MTNKLTQPKGSVSKQLNIQTISRICGCNENEVSYIYNQLDIFNIKYLYDPISEKICISNG--SGLVLSYTVSENSIMVIT
  consensus/100%                MsNKLTQPKGSlSKphNhpsIuRlhGhpcstVuYI.spl.I.shphLYD..oEp.Ch.pG..oGhslSahlopsSlhl.T
```

K

```
                    cov    pid    1      .         .         .         .         :         .         .         .
1 YP_009153198.1 100.0% 100.0%     MAFKFKGSLSAVDATLKGGVKFDNEKLPDSKNMPEGKFTINEIDNSSTTDLASNTLVTNFGKQDNVAGMLSFNTGDWENN
2 YP_007007683.1  87.2%  48.1%     MAFKFKGSLSAVDATLKGGVKFDNEKLPDSKNMPEGKFTINEIDNSSTTDIASNTLVTNFGKPDNVAGMLSFNTGDWENN
  consensus/100%                   MAFKFKGSLSAVDATLKGGVKFDNEKLPDSKNMPEGKFTINEIDNSSTTDlASNTLVTNFGK.DNVAGMLSFNTGDWENN

                    cov    pid    81     .         1         .         .         .         .         :         .
1 YP_009153198.1 100.0% 100.0%     NSAPVKLMMRVVNNSNDSDWVTIFDSTKTTVTWSSIASAGQTVFTVPNLDFKKAIIYINGILQYPGISYQTFGGTVTFSN
2 YP_007007683.1  87.2%  48.1%     NSAPVKLMMRVVNNNNDSDWVTIFDSSKTTVTWSSVASAGQSVFTVPNLDFKKAIIYINGILQYPGISYQTFGGTVTFSN
  consensus/100%                   NSAPVKLMMRVVNNsNDSDWVTIFDSoKTTVTWSSlASAGQoVFTVPNLDFKKAIIYINGILQYPGISYQTFGGTVTFSN

                    cov    pid    161    .         .         .         2         .         .         .         .
1 YP_009153198.1 100.0% 100.0%     SLQAGDNVYIVVGEDSDNTTNTQYISTALDNQTTIILPYEKNSNYLFINGVLQYPNSFTIVNNTITLNSSMQQDDNIFVL
2 YP_007007683.1  87.2%  48.1%     YLQSGDNVYIVVGKDSDNTTNIQYISTATTEQNTITLPYSRSSNYVYINGILQYPSTFTISNDIITLNSSMQEGDNIFVL
  consensus/100%                   .LQuGDNVYIVVGcDSDNTTNhQYISTAhspQsTIhLPYp+sSNYlaINGlLQYPsoFTIsNshITLNSSMQpsDNIFVL
```

```
                                  cov     pid    241
                                                          :         .         .         .         .         3         .         .
1 YP_009153198.1 100.0% 100.0%         SNDKIVPNYTAVAIQDQTDFTITGTIIDPVVYINGVLQYDTHYTISGNTLSFVSKLFAGDEVLVFLDSLNLIDNINTEYN
2 YP_007007683.1  87.2%  48.1%         SNDKSVSNYTSVAVQDQTDFTISGTIVEPTVYVNGVLQYDDYYNISGNTLSFVGKLFEGDQVLVFLDNPQLIDDVNTEYT
  consensus/100%                       SNDK.VsNYTuVAlQDQTDFTIoGTIl-PsVYlNGVLQYDsaYsISGNTLSFVuKLFtGDpVLVFLDs.pLIDslNTEYs

                                  cov     pid    321
                                                          .         .         :         .         .         .         .         4
1 YP_009153198.1 100.0% 100.0%         SVIDDVNNSNKINIPYFHFSELQVFINGILQNPDNGAYDLNGTEVTLSSPLQEGDDIHVIVYNSPIQDDNIVTKADLSSY
2 YP_007007683.1  87.2%  48.1%         NIIDDTNNSNKINIPYFHFSELQVFINGILQNPDNGAYDLNGTEVTLSSPLQEGDDIHVIVYNSPIQDDNIVTKADLSSY
  consensus/100%                       slIDDsNNSNKINIPYFHFSELQVFINGILQNPDNGAYDLNGTEVTLSSPLQEGDDIHVIVYNSPIQDDNIVTKADLSSY

                                  cov     pid    401
                                                          .         .         .         .         :         .         .         .
1 YP_009153198.1 100.0% 100.0%         ASITELNLLKDSLKAEGINLKWQPHLPSIEVAFGLPRRSLKIWKAGNTSTINQYWLYPVDGTVWSGIGTLGNVPSTPFYK
2 YP_007007683.1  87.2%  48.1%         ASINELNLLKDSLKTEGINLKWQPHLPSIEVAFGLPRRSLKIWKAGNTSTANQYWLYPVDGTVWAGVGILGTVPDVPFYK
  consensus/100%                       ASIsELNLLKDSLKsEGINLKWQPHLPSIEVAFGLPRRSLKIWKAGNTSThNQYWLYPVDGTVWuGlGhLGsVPssPFYK

                                  cov     pid    481
                                                          .         5         .         .         .         .         :         .
1 YP_009153198.1 100.0% 100.0%         LDSNKDVITWTYTATSDNINRIFVPYNFGSINIFINGVLQNIELGHYTYNGQYIDLNGSLNIGDNLVAILGKLILNTNPY
2 YP_007007683.1  87.2%  48.1%         LDSNKDVITWTYTATSDNINRIFVPYNFGSINIFINGVFQSVELGHYTYTGQYINLNGALQTGDNLIAILGKLILNTNPY
  consensus/100%                       LDSNKDVITWTYTATSDNINRIFVPYNFGSINIFINGVhQslELGHYTYsGQYIsLNGuLphGDNLlAILGKLILNTNPY

                                  cov     pid    561
                                                          .         .         .         6         .         .         .         .
1 YP_009153198.1 100.0% 100.0%         LTYETANGQFVSKSDLSSNTGSLMVGTSDGNNLQQTLNNKVNSVL--LS--SNNGSSIVGTSSGLSVQAEIDNIKKQFTD
2 YP_007007683.1  87.2%  48.1%         LTIESA-SKFITKSDIYNTDGAEKIGTSDGRNVQINLNEVSDFNKNLESNNQNLGANLINTEIDISVG--------KWIK
  consensus/100%                       LThEoA.upFloKSDl.sssGu.hlGTSDGpNlQ.sLNphss.sh...S..pN.GusllsTp.slSVt........pahc
```

L

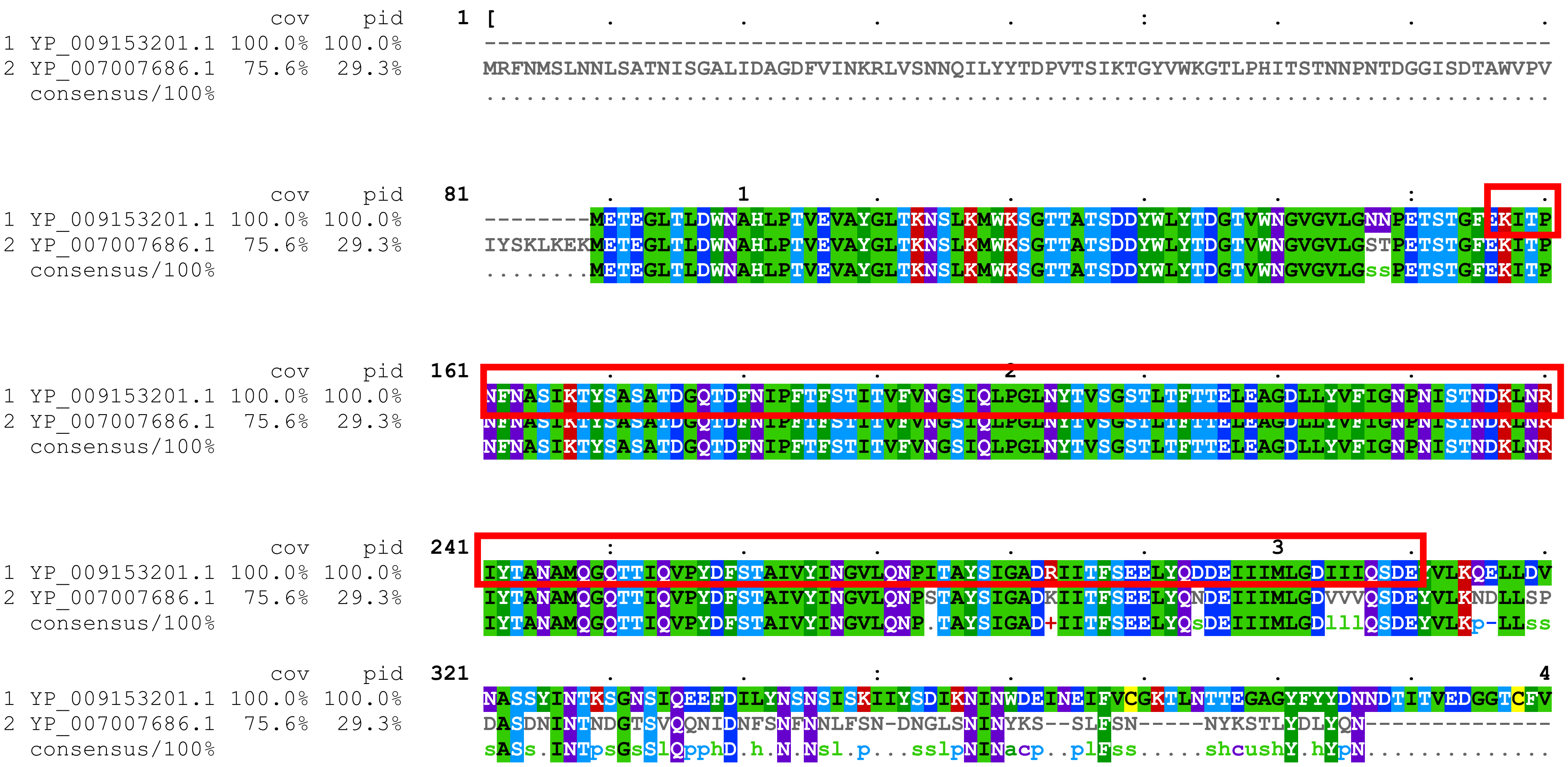

cov pid 1 [. . . . : . . .
1 YP_009153201.1 100.0% 100.0% --
2 YP_007007686.1 75.6% 29.3% MRFNMSLNNLSATNISGALIDAGDFVINKRLVSNNQILYYTDPVTSIKTGYVWKGTLPHITSTNNPNTDGGISDTAWVPV
consensus/100% ....
cov pid 81 . 1 . . . . : .
1 YP_009153201.1 100.0% 100.0% --------METEGLTLDWNAHLPTVEVAYGLTKNSLKMWKSGTTATSDDYWLYTDGTVWNGVGVLGNNPETSTGFEKITP
2 YP_007007686.1 75.6% 29.3% IYSKLKEKMETEGLTLDWNAHLPTVEVAYGLTKNSLKMWKSGTTATSDDYWLYTDGTVWNGVGVLGSTPETSTGFEKITP
consensus/100% ........METEGLTLDWNAHLPTVEVAYGLTKNSLKMWKSGTTATSDDYWLYTDGTVWNGVGVLGssPETSTGFEKITP
cov pid 161 . . . 2 . . . .
1 YP_009153201.1 100.0% 100.0% NFNASIKTYSASATDGQTDFNIPFTFSTITVFVNGSIQLPGLNYTVSGSTLTFTTELEAGDLLYVFIGNPNISTNDKLNR
2 YP_007007686.1 75.6% 29.3% NFNASIKTYSASATDGQTDFNIPFTFSTITVFVNGSIQLPGLNYTVSGSTLTFTTELEAGDLLYVFIGNPNISTNDKLNR
consensus/100% NFNASIKTYSASATDGQTDFNIPFTFSTITVFVNGSIQLPGLNYTVSGSTLTFTTELEAGDLLYVFIGNPNISTNDKLNR
cov pid 241 : . . . . 3 . .
1 YP_009153201.1 100.0% 100.0% IYTANAMQGQTTIQVPYDFSTAIVYINGVLQNPITAYSIGADRIITFSEELYQDDEIIIMLGDIIIQSDEYVLKQELLDV
2 YP_007007686.1 75.6% 29.3% IYTANAMQGQTTIQVPYDFSTAIVYINGVLQNPSTAYSIGADKIITFSEELYQNDEIIIMLGDVVVQSDEYVLKNDLLSP
consensus/100% IYTANAMQGQTTIQVPYDFSTAIVYINGVLQNP.TAYSIGAD+IITFSEELYQsDEIIIMLGDlllQSDEYVLKp-LLss
cov pid 321 . . : 4
1 YP_009153201.1 100.0% 100.0% NASSYINTKSGNSIQEEFDILYNSNSISKIIYSDIKNINWDEINEIFVCGKTLNTTEGAGYFYYDNNDTITVEDGGTCFV
2 YP_007007686.1 75.6% 29.3% DASDNINTNDGTSVQQNIDNFSNFNNLFSN-DNGLSNINYKS--SLFSN-----NYKSTLYDLYQN--------------
consensus/100% sASs.INTpsGsSlQpphD.h.N.Nsl.p...sslpNINacp..plFss.....shcushY.hYpN..............

M

```
                      cov     pid     1 [         .         .         .         .         :         .         .         .
1 YP_009153202.1 100.0% 100.0%        MSLSNLSSSNSNGAFLDYGEFRSGVYIQTSNQILYFTDENGNKVGYQWKGLLPHTTTTNDPSTDGGISDTAWCSIVGSGF
2 YP_007007687.1  69.5%  31.4%        MSLSNLSSSNSNGAFLDYGEFRSGVYIQTSNQILYFTDENGNKVGYQWKGLLPHTTTTNDPNTDGGISDTAWCSIVGSGF
  consensus/100%                      MSLSNLSSSNSNGAFLDYGEFRSGVYIQTSNQILYFTDENGNKVGYQWKGLLPHTTTTNDPsTDGGISDTAWCSIVGSGF

                      cov     pid    81           .         1         .         .         .         .         :         .
1 YP_009153202.1 100.0% 100.0%        IEKLSKEGIDLSWKAHLPTVEVSYNLPHKSLKIWEEGTNSTDNDYWLYPGDGTVWNGIGVLGDIPDAPFKQIVPQNNVIE
2 YP_007007687.1  69.5%  31.4%        IEKLSKEGIDLSWKAHLPTVEVSYNLPHKSLKIWEEGTISTDNDYWLYPGDGTVWNGVGVLGSIPDVPFKQIVPQNNVIE
  consensus/100%                      IEKLSKEGIDLSWKAHLPTVEVSYNLPHKSLKIWEEGT.STDNDYWLYPGDGTVWNGlGVLGsIPDsPFKQIVPQNNVIE

                      cov     pid   161           .         .         .         2         .         .         .         .
1 YP_009153202.1 100.0% 100.0%        WSAIATEGQNQFTVPYEFTNISVFINGLLQNKSTGGYVVNGSTVTLNGSLKAGDDIHVVISNIPIKNINYITDVE-----
2 YP_007007687.1  69.5%  31.4%        WSTIATEGQNQFTVPYEFTNISVFINGLLQNKSTGGYVVNGSTVTLNGSLKAGDVIHVVISNVHTTNIIYALQSDLNNYY
  consensus/100%                      WSsIATEGQNQFTVPYEFTNISVFINGLLQNKSTGGYVVNGSTVTLNGSLKAGDsIHVVISNl.hpNI.Yhhps-.....

                      cov     pid   241           :         .         .         .         .         3         .         .
1 YP_009153202.1 100.0% 100.0%        ----LSQPDAAQKIGLLHGGNIQNLQNFLSFDMFNIDKTGST--DVTSQINNIFSLANQLNIPIKQHDGTYLVSGSTIFT
2 YP_007007687.1  69.5%  31.4%        TKTNLNSTSGSSYIGLTQGGTLDEAITYITPQMVNDTPYQTFGTALTSACNKA--MANGIG-EVRIPAGRYILDKTVDIS
  consensus/100%                      ....LspssuuphIGLhpGGslpph.salo.pMhN.s.htoh..slTSthNph..hANtls..l+..sGpYllstos.ho
```

N

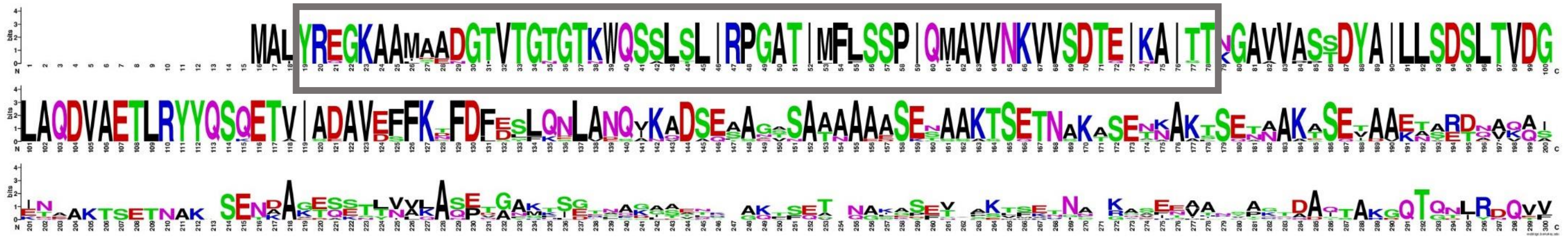

Figure S1. Conserved sequences at the N-terminus of the *Klebsiella* RBPs per family using a WebLogo when multiple RBP sequences are available, or a pairwise alignment when only two RBP sequences are available. (A) N-terminal anchor of the first RBP of KP32viruses; (B) conserved peptide of the second RBP of KP32viruses; (C) N-terminal anchor of the first RBP of KP34viruses; (D) conserved peptide of the second RBP of KP34viruses; (E) N-terminal anchor of the first RBP of viruses belonging to the Menlow group; (F) conserved peptide of the second RBP of viruses belonging to the Menlow group; (G) conserved peptide of the third RBP of viruses belonging to the Menlow group; (H) conserved peptide of the fourth RBP of viruses belonging to the Menlow group; (I) conserved peptide of the second RBP of viruses belonging to the ΦK64-1; (J) conserved peptide of the fourth RBP of viruses belonging to the ΦK64-1 group; (K) anchor of the fifth RBP of viruses belonging to the ΦK64-1 group; (L) conserved peptide of the eighth RBP of viruses belonging to the ΦK64-1 group; (M) conserved peptide of the ninth RBP of viruses belonging to the ΦK64-1 group; (N) N-terminal anchor of the first RBP of KP36viruses. The red frames indicate the region of RBPs detected to be homologous to T4gp10. In the case of KP36viruses, the region homologous to the T5 distal tail protein domain B was indicated with grey frame.
