# Supplementary material for: Modeling the Architecture of Depolymerase-Containing Receptor Binding Proteins in Klebsiella Phages
Source: Front Microbiol. 2019 Nov 15;10:2649. doi: 10.3389/fmicb.2019.02649 (PMC6872550; doi:10.3389/fmicb.2019.02649)
Supplement: Supplementary file 2 [file Table_1.pdf]

Supplementary materials

Table S1. List of phages analysed in this work, belonging to particular groups: A – KP32viruses, B – KP34viruses, C - JD001 group, D – Menlow group, E - ΦK64-1 group, F - KP36viruses

A

Phage	Accesion numer	Genome length	Classification
KP32	NC_013647.1	41119 bp	Podoviridae, Autographivirinae, Kp32virus
K11	NC_011043.1	41181 bp	Podoviridae, Autographivirinae, Kp32virus
KP32 isolate 194	MH172262.1	41161 bp	Podoviridae, Autographivirinae
KpV763	KX591654.1	40765 bp	Podoviridae, Autographivirinae, unclassified Kp32virus
KP32 isolate 192	MH172261.1	40635 bp	Podoviridae, Autographivirinae
K5-2	KY389315.1	41116 bp	Podoviridae, Autographivirinae, unclassified Kp32virus
KP32 isolate 196	MH172264.1	40337 bp	Podoviridae, Autographivirinae
KpV766	KX712071.1	41283 bp	Podoviridae, Autographivirinae, unclassified Kp32virus
KpV289	NC_028977.1	41054 bp	Podoviridae, Autographivirinae, Kp32virus
IME 205	KU183006.1	41310 bp	Podoviridae, Autographivirinae, unclassified Kp32virus
K5	NC_028800.1	41698 bp	Podoviridae, Autographivirinae, Kp32virus
KpV767	KX712070.1	40395 bp	Podoviridae, Autographivirinae, unclassified Kp32virus
2044-307w	MF285615.1	40048 bp	Podoviridae, Autographivirinae, unclassified Kp32virus
K5-4	KY389316.1	40163 bp	Podoviridae, Autographivirinae, unclassified Kp32virus
IL33	KY652724.1	41335 bp	Podoviridae, Autographivirinae, unclassified Kp32virus
PRA33	KY652723.1	40605 bp	Podoviridae, Autographivirinae, unclassified Kp32virus
BIS33	KY652725.1	41697 bp	Podoviridae, Autographivirinae, unclassified Kp32virus
IME321	MH587638.1	39906 bp	Podoviridae; Autographivirinae, unclassified Kp32virus
KP32 isolate 195	MH172263.1	40540 bp	Podoviridae, Autographivirinae
SH-Kp 152410	MG835568.1	40945 bp	Podoviridae, Autographivirinae, unclassified Kp32virus
Kp1	NC_028688.1	40114 bp	Podoviridae, Autographivirinae, Kp32virus

B

Phage	Accesion number	Genome length	Classification
KP34	NC_013649.2	43809 bp	Podoviridae, Autographivirinae, Kp34virus
SU503	NC_028816.1	43809 bp	Podoviridae, Autographivirinae, Kp34virus
F19	NC_023567.2	43766 bp	Podoviridae, Autographivirinae, Kp34virus
KpV475	NC_031025.1	42201 bp	Podoviridae, Autographivirinae, Kp34virus
KpV71	NC_031246.1	43267 bp	Podoviridae, Autographivirinae, Kp34virus
NTUH-K2044-K1-1	NC_025418.1	43871 bp	Podoviridae, Autographivirinae, Kp34virus
KPV811	KY000081.1	42641 bp	Podoviridae, Autographivirinae, unclassified Kp34virus
KpV48	KX237514.1	44623 bp	Podoviridae, Autographivirinae, unclassified Kp34virus
phiBO1E	KM576124.1	43865 bp	Podoviridae, Autographivirinae, unclassified Kp34virus
AltoGao	MF612071.1	43012 bp	Podoviridae, Autographivirinae, unclassified Kp34virus
Kp2	NC_028664.1	43963 bp	Podoviridae, Autographivirinae, Kp34virus
KpV74	KY385423.1	44094 bp	Podoviridae, Autographivirinae, unclassified Kp34virus
SU552A	NC_028870.1	43595 bp	Podoviridae, Autographivirinae, Kp34virus
KpV41	NC_028670.1	44203 bp	Podoviridae, Autographivirinae, Kp34virus
phiKpS2	MG835568.1	40945 bp	Podoviridae, Autographivirinae, unclassified Kp34virus
KP-Rio/2015	MG835568	40945 bp	Podoviridae, Autographivirinae, unclassified Kp34virus
myPSH1235	MG972768.1	45135 bp	Podoviridae, Autographivirinae, unclassified Kp34virus

C

Phage	Accesion number	Genome length	Classification
JD001	NC_020204.1	48814 bp	Myoviridae
KpV52	KX237516.1	47405 bp	Myoviridae, unclassified Msw3virus
KpV79	MF663761.1	47760 bp	Myoviridae, unclassified Msw3virus

D

Phage	Accesion number	Genome length	Classification
Menlow	MG428990.1	157281 bp	Ackermannviridae, unclassified Ackermannviridae
KpS110	MG770379.1	156801 bp	Ackermannviridae, unclassified Ackermannviridae
May	MG428991.1	159631 bp	Ackermannviridae, unclassified Ackermannviridae
0507-KN2-1	NC_022343.1	159991 bp	Ackermannviridae, unclassified Ackermannviridae

E

Phage	Accesion number	Genome length	Classification
ΦK64-1	NC_027399.1	346602 bp	Myoviridae, unclassified Myoviridae
RaK2	NC_019526.1	345809 bp	Myoviridae, unclassified Myoviridae

F

Phage	Accesion number	Genome length	Classification
KP36	NC_029099.1	49797 bp	Siphoviridae, Tunavirinae, Kp36virus
KLPN1	NC_028760.1	49037 bp	Siphoviridae, Tunavirinae, Kp36virus
KOX1	KY780482.1	50526 bp	Siphoviridae, Tunavirinae, Kp36virus, unclassified Kp36virus
JY917	MG894052.1	37655 bp	Siphoviridae, Tunavirinae, Kp36virus, unclassified Kp36virus
1513	NC_028786.1	49462 bp	Siphoviridae, Tunavirinae, Kp36virus
Sushi	NC_028774.1	48754 bp	Siphoviridae, Tunavirinae, Kp36virus
NJS1	MH445453.1	49292 bp	Siphoviridae, Tunavirinae, Kp36virus, unclassified Kp36virus
MezzoGao	MF612072.1	49807 bp	Siphoviridae, Tunavirinae, Kp36virus, unclassified Kp36virus
GML-KpCol1	MG552615.1	50249 bp	Siphoviridae, Tunavirinae, Kp36virus, unclassified Kp36virus
PKP126	NC_031053.1	50934 bp	Siphoviridae, Tunavirinae, Kp36virus
KpV522	KX237515.1	51099 bp	Siphoviridae, Tunavirinae, Kp36virus, unclassified Kp36virus
KPN N141	MF415412.1	49090 bp	Siphoviridae, Tunavirinae, Kp36virus, unclassified Kp36virus
